# Combining pMINFLUX, graphene energy transfer and DNA-PAINT for nanometer precise 3D super-resolution microscopy

**DOI:** 10.1038/s41377-023-01111-8

**Published:** 2023-03-10

**Authors:** Jonas Zähringer, Fiona Cole, Johann Bohlen, Florian Steiner, Izabela Kamińska, Philip Tinnefeld

**Affiliations:** 1 Department of Chemistry and Center for NanoScience, Ludwig-Maximilians-Universität München, Butenandtstr. 5-13 Haus E, 81377 München, Germany; 2 Institute of Physical Chemistry Polish Academy of Sciences, Kasprzaka 44/52, 01-224, Warsaw, Poland; 3 Present Address: Department of Physics, Ludwig-Maximilians-Universität München, Schellingstraße 4, 80799 München, Germany

**Keywords:** Super-resolution microscopy, Biophotonics, Imaging and sensing

## Abstract

3D super-resolution microscopy with nanometric resolution is a key to fully complement ultrastructural techniques with fluorescence imaging. Here, we achieve 3D super-resolution by combining the 2D localization of pMINFLUX with the axial information of graphene energy transfer (GET) and the single-molecule switching by DNA-PAINT. We demonstrate <2 nm localization precision in all 3 dimension with axial precision reaching below 0.3 nm. In 3D DNA-PAINT measurements, structural features, i.e., individual docking strands at distances of 3 nm, are directly resolved on DNA origami structures. pMINFLUX and GET represent a particular synergetic combination for super-resolution imaging near the surface such as for cell adhesion and membrane complexes as the information of each photon is used for both 2D and axial localization information. Furthermore, we introduce local PAINT (L-PAINT), in which DNA-PAINT imager strands are equipped with an additional binding sequence for local upconcentration improving signal-to-background ratio and imaging speed of local clusters. L-PAINT is demonstrated by imaging a triangular structure with 6 nm side lengths within seconds.

## Abstract (web summary)

Super-resolution microscopy with 3 nm resolution and sub-nanometer localization precision is achieved on DNA origami structures with DNA PAINT and L-PAINT, a novel scheme for fast super-resolution.

## Graphical abstract

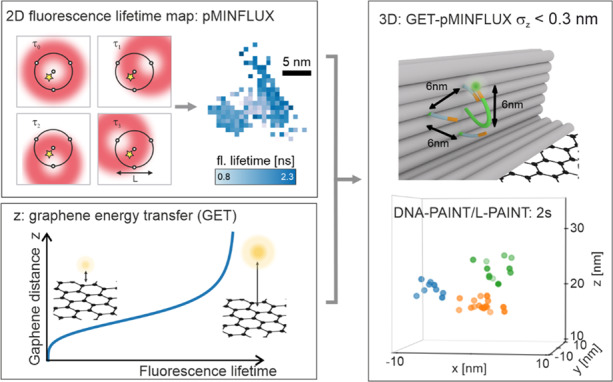

## Introduction

3D super-resolution with nanometer precision opens exciting new insights in nanostructures and biological systems by achieving molecular or even submolecular resolution. There is a multitude of techniques extending single-molecule localization microscopy (SMLM) to the third dimension, including PSF manipulation^1,2^, 4-Pi microscopy^3^, total internal reflection fluorescence (TIRF) microscopy^4^, repetitive optical selective exposure (ROSE-Z)^5^ or Supercritical Angle Localization Microscopy (SALM)^6^ and many more. However, in these techniques, the precision is mostly limited to the emission information, and hence the camera localization does not reach precisions of about the size of a fluorophore of 1–2 nm of all three dimensions. The coordinate-targeted approach of 3D stimulated emission depletion microscopy (STED)^7^ has similar limitations in precision. To this end MINFLUX nanoscopy^8^ and later MINSTED nanoscopy^9^ were introduced. By interrogating the emitter location with a series of targeted illuminations, localization precisions of <2 nm are reached with moderate photon budgets. It later was extended to 3D by superimposing vortex beams to generate a tophat^10^. However, the instrumental and engineering requirements increase with dimensionality and the photon budget is divided between the axial and lateral dimensions. Each photon only contributes to either the lateral or the axial localization depending on the kind of vortex mask of the respective illumination event.

Alternative to optical approaches, the axial position of a fluorescent dye can be determined from near-field interactions with a modified coverslip. To this end, energy transfer between a dye and a metal- or graphene-layer is read out from fluorescence intensity or fluorescence lifetime and is converted to an axial information in approaches termed metal-induced energy transfer (MIET)^11–13^ or graphene energy transfer (GET)^14–16^. GET with graphene-on-glass coverslips has the advantage of high optical substrate transparency (>97%)^17^, lack of autofluorescence and steep d^−4^ distance dependence yielding the highest localization precision within its dynamic range^14,18^.

In this work, we combine GET and pulsed-interleaved MINFLUX nanoscopy (pMINFLUX) with DNA-PAINT to enable nanometer precise 3D super-resolution imaging. pMINFLUX was introduced as simpler MINFLUX realization that additionally provides the fluorescence lifetime^19^. In combination with GET, axial position determination from the intensive property fluorescence lifetime is advantageous as it is intensity independent and does not require internal referencing. In the GET-pMINFLUX combination, each photon is synergetically used for both, xy- as well as z-localization optimally exploiting the available information^20^. Using DNA origami nanopositioners, fluorescent molecules and DNA point accumulation for imaging in nanoscale topography (DNA-PAINT), binding sites are placed precisely in 3D^21^. These nanopositioners are then used to evaluate the GET-pMINFLUX DNA-PAINT combination for 3D localization and 3D super-resolution imaging at different distances to graphene^14^. To overcome the comparatively small field of view of pMINFLUX and the limited binding kinetics of DNA-PAINT, we also introduce local PAINT (L-PAINT) in which a DNA imager strand binds for longer times locally and quickly probes (“PAINT”) neighboring binding sites.

## Results

**Fig. 1:**
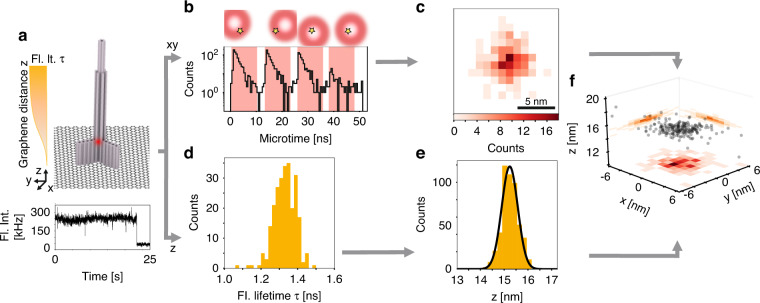
Combining pMINFLUX with graphene energy transfer for precise 3D localizations. **a** Top: Schematic of a DNA origami structure with a single dye positioned at a height of 16 nm above a graphene-on-glass coverslip. Bottom: Fluorescence intensity trace of the total fluorescence intensity of a single dye molecule in a single DNA origami structure. **b** Fluorescence decays for each of the four pulsed interleaved vortex-shaped beams which are focused on the sample arranged in a triangular pattern with the fourth beam placed at the center of the triangular structure. The star indicates the xy position of the dye molecule. **c** xy localization histogram of time bins. **d** Distribution of fluorescence lifetimes obtained from the time bins. **e** Distribution of the distances to graphene z calculated from the fluorescence lifetimes of d). **f** 3D localizations of the full fluorescence intensity trace using the 2D information of pMINFLUX and the z distances from the fluorescence lifetimes. The individual localizations are shown in black and on the sides the corresponding projections with a binning of 1 nm for xy and 0.2 nm for z

In GET-pMINFLUX nanoscopy, the xy position of a single fluorescent molecule placed on a graphene-on-glass coverslip using a DNA origami nanopositioner (Fig. 1a, top) is localized using pMINFLUX nanoscopy, while the axial position is determined by GET. To determine the 2D position of the dye it is excited by four spatially displaced and pulsed interleaved vortex beams^19^. By binning the fluorescent intensity trace (Fig. 1a, bottom), the number of photons corresponding to each of the four pulsed vortex beams is extracted via time-correlated single-photon-counting (TCSPC) (Fig. 1b). The position of the fluorophore is determined by a maximum likelihood estimator as described in earlier works^8,19^ for the fluorescence intensities and the known excitation profile and positions of all beams. By dividing the fluorescence intensity trace into time or photon bins, the same molecule is localized many times yielding 2D histograms of localizations (Fig. 1c).

For the axial dimension, the pulsed interleaved approach entails the fluorescence lifetime of the molecule, which is extracted for each localization from the TCSPC histogram (Fig. 1d and Supplementary Information 2.1). In the case of a designed 16 nm distance to graphene, the resulting fluorescence lifetime is 1.3 ns. With an unquenched fluorescence lifetime of ATTO647N of 4.2 ns, a GET efficiency of 69% is measured. With the known *d*^−4^ fluorescence lifetime – distance to graphene relation^14,18^, the distance to graphene z is calculated for each localization (Fig. 1e). The resulting distance to graphene z of 15.3 nm is obtained using the 50% energy transfer distance d_0_ of 18.5 nm for ATTO647N^14^. By combining both, the 2D position and the distance to graphene z, a single fluorophore is localized in 3D with a precision of 1.9 nm in lateral and 0.3 nm in axial dimensions with moderate photon count rates of 1000 photons (Fig. 1f). Notably in contrast to the xy localization, where a nanometer precise drift correction is needed, the z localization is not impeded by drift, as the distance to graphene is measured. This is another reason for the remarkable precision in z-direction.

The precision in z is, on the one hand, determined by the precision of the fluorescence lifetime estimation, on the other hand, dependent on the slope of the graphene energy transfer relation hence on the absolute distance to graphene z (Supplementary Information 2.3).

**Fig. 2:**
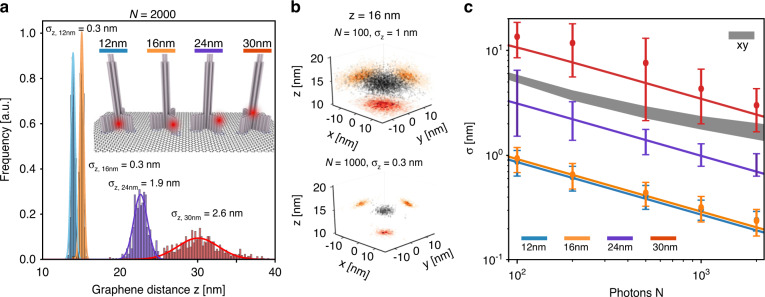
Localization performance of the GET p-MINFLUX combination. **a** Distributions of the distance z from graphene for exemplary molecules with a different dye position ranging from 12 to 30 nm analyzed with a fixed number of photons of *N* = 2000. **b** Exemplary 3D localization plots of a single fixed dye at a distance to graphene z of 16 nm with different number of photons, N, used to evaluate the precision. In this measurement, the SBR was 7. The individual localizations are shown in black and on the sides the corresponding projections. The red histogram is projection to xy. The orange histograms are the projection to xz and yz. The individual localizations are shown in black and on the sides the corresponding projections have a binning of 1 nm for xy and 0.2 nm for z. **c** Localization precision in xy and z as a function of the number of photons N for fixed dye molecules placed at different height. The gray stripe indicates the xy precision of MINFLUX in the corresponding experiments, the lines indicate the theoretical lower limit of precision

To evaluate the dependency of the absolute distance to graphene z on the precision, fluorophores were placed at different heights using DNA origami nanopositioners (Fig. 2a). Close to the 50% quenching distance d_0_, GET reaches axial precisions of <0.3 nm at moderate photon number of 2000 photons per localization outperforming the xy precision of pMINFLUX nanoscopy which is at 1–2 nm. At higher distances to graphene of around 30 nm, the axial GET precision matches the lateral precision of pMINFLUX.

As also the axial precision is a function of the number of detected photons (N), the fluorescence intensity trace of a fixed ATTO 647 N fluorophore is binned using different bin widths, hence different photon numbers N ranging from 100 to 2000 photons (Fig. 2b). For a 16 nm distance to graphene and N ranging from 100 to 1000, the resulting axial precision is estimated between 1 and 0.3 nm, respectively. The comparison of the axial precision for different heights in dependence of the photon number is depicted in Fig. 2c and compared to the photon number dependent precision of the xy determination by MINFLUX. Both the axial as well as the lateral precision show the expected dependence. The theory of the axial precision agrees well within the error of the experimental data (Supplementary Information 2.3). A suitable range for precisions of GET and pMINFLUX is between 8 and 35 nm (Supplementary Information Fig. S4). At higher distances to graphene, the slope of the energy transfer relation decreases and the axial precision drops. Due to the excitation with vortex shaped laser beams, a challenge of MINFLUX is the Signal to Background Ratio (SBR)^10^. Hence below ~8 nm distance to graphene, the signal is so strongly quenched that the SBR drops and MINFLUX localizations are less precise.

For MINFLUX nanoscopy, redox blinking or thiol induced switching was used to enable successive localization of single molecules as a background signal from diffusing molecules required in PAINT approaches is avoided^8,10^. This, however, limits the choice of dyes, the duty cycle and the available photon budget. To apply GET-pMINFLUX in combination with photon optimized DNA-PAINT^22^, we increase the binding kinetics by a concatenated and periodic DNA motif^23^ such that a DNA-PAINT imager has multiple binding options.

**Fig. 3:**
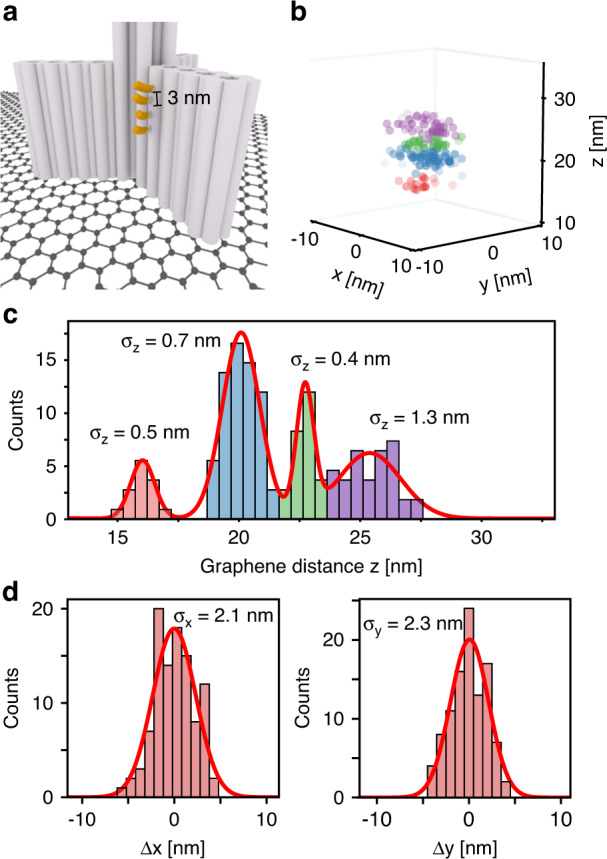
Super-resolution DNA-PAINT imaging with pMINFLUX and GET. **a** Schematic illustration of the imaged structure with protruding docking sites. **b** 3D localizations of the DNA-PAINT trace with corresponding projections on the side. Binning along the *x* and *y* axis is 1.5 nm and along the *z* axis 0.5 nm. **c** Histogram of the distance to graphene z of the selected xy localizations. **d** Histograms of xy localizations relative to the mean molecule position

Using a 7-nucleotides long ATTO542 labeled DNA-PAINT imager strand, a 3D docking site pattern on a DNA origami nanopillar was imaged, of which the central motif is depicted in Fig. 3a and the full structure is shown in Fig. S5. Out of the 300 s of the GET-pMINFLUX measurement, a 3D localization map was generated (Fig. 3b). To evaluate the performance of GET-pMINFLUX with DNA-PAINT, a projection of the localizations representing the four docking sites in the center of the structure (as depicted in Fig. 3a) on the z-axis is shown in Fig. 3c. A multi-Gaussian fit reveals the well-resolved 3 nm distances between the docking sites with axial precisions between 0.4 and 1.3 nm. With xy precisions of the individual docking sites between 1.6 and 2.3 nm, we show nanometer precise 3D super-resolution resolving a 3D structure with 3-nm features. Beside enabling 3D super-resolution, the efficient energy transfer to graphene eliminates background localization events as unspecific bound imagers are fully quenched close to graphene. This is especially important for MINFLUX nanoscopy, which is prone to background influences from unspecific binding events in the vicinity of the vortex beams.

In DNA-PAINT, imaging speed requires higher imager concentration^23–25^. However, a higher imager concentration reduces the SBR as diffusing molecules are excited by the comparable large excitation volumes of MINFLUX. The situation is aggravated by the serial nature of MINFLUX, which calls for fast binding kinetics to speed up imaging and to reduce the requirements for drift corrections.

**Fig. 4:**
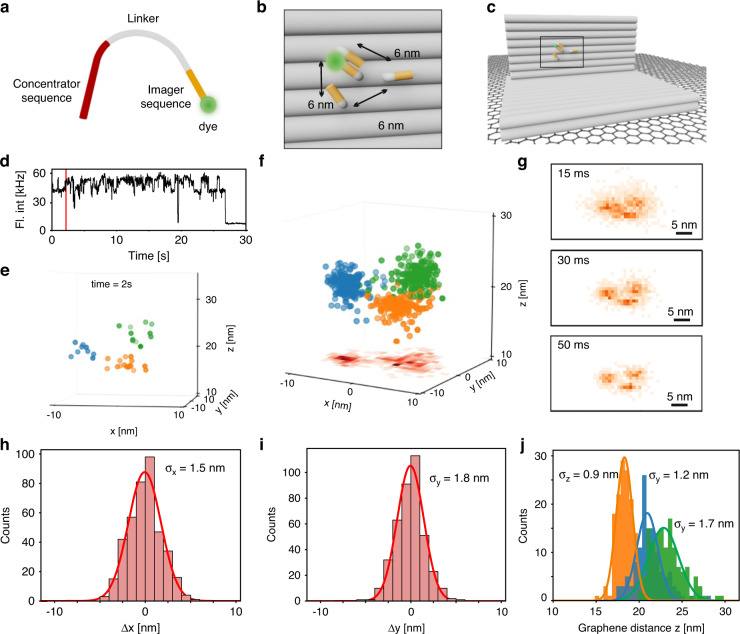
L-PAINT imaging with pMINFLUX and GET. **a** Schematic of a L-PAINT imager strand. **b** A DNA origami structure with 3 protruding strands to which a Cy3b dye-labeled imager sequence can transiently bind. The L-PAINT imager is bound to the DNA origami structure by the concentrator sequence. **c** Zoom out of the DNA origami structure on graphene. **d** Fluorescence intensity trace with a red line at 2 s. **e** 3D localizations of the L-PAINT trace after 2 s. **f** 3D localizations of the 30 s L-PAINT trace with the xy projection on the bottom with a binning of 1 nm. **g** 2D histogram of the xz projection with a binning of 1 nm with time bins for localization varying from 15 to 50 ms. **h** Histogram of distances to each mean binding position in x. **i** Histogram of distances to each mean binding position in y. **j** Histogram of the distances to graphene z

We address this challenge by local PAINT (L-PAINT), in which a locally high imager concentration is achieved by a hierarchy of binding interactions without creating higher background. In L-PAINT, the imager strand has two binding sequences (Fig. 4a). One binding site is comparatively strong (in the extreme, it can be thermally stable) and keeps the imager strand bound to the structure of interest creating a locally high concentration (therefore referred to as concentrator sequence). The labeled imager sequence on the other end of the L-PAINT imager creates the short binding events with docking strands typical for DNA-PAINT. The size of the docking site cluster that can be sampled with one imager strand binding event depends on the length of the linker between concentrator sequence and imager sequence.

For L-PAINT demonstration, we design a L-PAINT imager whose concentrator sequence binds stably to a DNA origami structure from which the imager sequence protrudes. In this limiting case of thermally stable binding of the imager strand over the time scale of the experiment, the local concentration of the imager strand is always high and no imager strand is required in the imaging buffer. The resulting imager concentration in solution thus corresponds to 0 nM. After a linker of 12 nucleotides, a Cy3B dye is attached at the end of the 7 nucleotides imager sequence (Fig. 4b). With GET-pMINFLUX, we visualize the imager sequence transiently binding to a triangular structure of docking strands protruding from the DNA origami structure displayed in Fig. 4c. Within only 2 s of the trace (Fig. 4d) and an integration time of 50 ms per localization, the triangular structure with 6 nm side length is resolved (Fig. 4e). After 30 sec, a highly sampled 3D localization map is obtained (Fig. 4f and extended data movie). Smaller integration times per localization show that the 6 nm jumps between the binding sites are already resolved at 15 ms integration time (Fig. 4g). Higher integration times show the trade-off between time resolution and localization precision (Fig. 4g).

To evaluate the performance, the localizations of the 30 s trace with 50 ms integration time were assigned to binding site clusters (Fig. 4f) in the xz projection, showing xy precisions of 1.5 and 1.8 nm (Fig. 4h, i) and axial precisions between 0.9 nm and 1.7 nm (Fig. 4j), respectively. In contrast to other super-resolution techniques, the dye is tracked at different binding sites; hence the trace is continuous resulting in a high number of localizations per unit time while avoiding high imager concentrations and double-binding events.

## Discussion

We present the combination of graphene energy transfer, pMINFLUX and DNA-PAINT. MINFLUX yields ultra-high localization precision in xy, in synergy GET provides outstanding z-localization close to the coverslip surface (8–35 nm) enabled by the fluorescence lifetime information of pulsed-interleaved MINFLUX, and DNA-PAINT provides the switching mechanism to proceed from super-localization to super-resolution. These three complementary and orthogonal components for super-resolution fully utilize the information of each detected photon and each component is realized fairly easily especially in light of recent progress^26,27^. Precisions better than 2 nm are shown in different experiments in all dimensions and structural details of 3 nm are resolved.

In order to increase binding kinetics and to reduce the background, we introduce L-PAINT with GET-pMINFLUX. The longer concentrator sequence keeps the imager strand connected to the region of interest while the scanning of the imager sequence quickly creates localizations in the proximity. With L-PAINT the imaging of docking sites with distances of 6 nm in 3D within less than 2 s and additionally, the tracking of the binding trajectory with 15 ms time resolution was demonstrated.

L-PAINT is not limited to DNA nanostructures and could also be applied to cell imaging with identical docking sites that are differently occupied by the concentrator and imager sequences. As fast imaging of the local environment circumvents drift problems L-PAINT is especially advantageous for dense molecular clusters. The limitation that the photon budget of the dye is distributed over different binding sites, which is less of a problem for MINFLUX than for less photon efficient camera based localization schemes could be compensated by slowly exchanging the imager strands with weakened concentrator sequences or by adding an additional binding hierarchy with a slowly exchanging dye labeled sequence for self-regenerating L-PAINT^28,29^.

In itself, GET-pMINFLUX is an extremely precise tool within a range of 8 to 35 nm above the coverslip. Here, the axial information is achieved by only adding a graphene layer on top of a coverslip. Furthermore, GET-pMINFLUX can be easily extended using spectral multiplexing^30^. In the future, GET-pMINFLUX nanoscopy will be used to investigate artificial bilayers^31^, cellular membranes and adhesion complexes as well as macromolecular complexes with nanometer 3D precision.

## Materials and methods

### Buffer

To stabilize all dyes, a combination of ROXS and oxygen scavenging system is used. Details of the buffers can be found in Table 1. For ATTO647N and Cy3B for the data in Figs. 1, 2, and 4, the buffer contains aqueous solution of aged Trolox^32^ with PCA (PCA/Trolox) and the second a 50× PCD (for measurements both buffers were mixed in a 1:50 ratio (50× PCD: Trolox/PCA).

**Table 1:** List of buffers with recipes

Buffer name	Recipe
PCA/Trolox	2 mM Trolox (6-hydroxy-2,5,7,8-tetramethylchroman-2-carboxylic acid) 12 mM PCA (protocatechuic acid) 12.5 mM MgCl_2_·6H_2_O 40 mM Tris base 20 mM acetic acid 1 mM EDTA-Na_2_·2H_2_O
PAINT PCA/Trolox	2 mM Trolox (6-hydroxy-2,5,7,8-tetramethylchroman-2-carboxylic acid) 2.4 mM PCA (protocatechuic acid) 6 mM MgCl_2_·6H_2_O 40 mM Tris base 20 mM acetic acid 1 mM EDTA-Na_2_·2H_2_O
50 × PCD	2.8 mM PCD (protocatechuate 3,4-dioxygenase from pseudomonas sp.) 50% glycerol 50 mM KCl 100 mM Tris HCl 1 mM EDTA-Na2·2H_2_O
FOB	12.5 mM MgCl_2_·6H_2_O 20 mM Tris base 20 mM acetic acid 1 mM EDTA-Na_2_·2 H_2_O

For DNA-PAINT experiments, the imaging buffer consists of an aqueous solution of aged Trolox with PCA (PAINT PCA/Trolox) and one of PCD. Both buffers were mixed in a 1(PCD):50(Trolox/PCA) ratio.

All chemicals were purchased from Sigma Aldrich.

### Preparation of DNA origami structures

The DNA origami structures were folded with 10-fold excess of oligonucleotide strands and a 100-fold excess of pyrene-modified oligonucleotides in comparison to the scaffold in 1× FOB buffer. Details of the folding program are found in ref. ^33^ After folding, 1× Blue Juice gel loading buffer was added to the folded DNA origami which was then purified via agarose-gel electrophoresis with 1.5% agarose gel in 50 mL of FOB buffer at 80 V for 1.5 h with 2 µL peqGREEN (ordered from VWR) per 100 µL buffer. The specific band for the nanostructure was extracted from the gel. Before putting the purified DNA origami solution onto graphene, the concentration was adjusted with FOB buffer to 75 pM.

### Graphene coverslips

In order to prepare graphene coverslips, a wet-transfer approach was used to transfer the CVD-grown graphene onto glass coverslips^14,16,27^. First, glass coverslips were cleaned with 1% Hellmanex and then subsequently washed twice in milliQ water, each step for 15 min in ultrasonication bath. Pieces of roughly 0.25 cm^2^ were cut from PMMA/graphene/copper foil and let to float on 0.2 M ammonium persulfate for copper etching. After ~3–4 h (when the copper foil was fully etched), PMMA/graphene was scooped gently with a clean coverslip and transferred to milliQ water to wash out the residues of ammonium persulfate. The washing step with the fresh milliQ water was repeated twice. Next, PMMA/graphene was scooped with a glass coverslip and carefully dried with a nitrogen stream. Samples were left for drying overnight. Next, ~10 μl of PMMA (M_w_ = 15,000 g/mol) in chlorobenzene (50 mg/mL) was drop-casted to cure the protection layer of PMMA on graphene. After ~30 min, when the solvent evaporated, the graphene-on-glass coverslip was placed in acetone for 7 min (×2) and in toluene for 7 min. After each step, the sample was dried with a nitrogen stream, and at last placed on active coal, heated on the heating plate to 230 C, for 30 min. Finally, the graphene-on-glass coverslip was removed from the active coal, and the incubation chamber (Grace Bio-Labs®) was placed on a glass coverslip so that the graphene piece was in the middle of the chamber.

### Sample preparation

The DNA origami solution was immobilized on graphene-on-glass coverslips for 2 min and then the sample was washed 3x using FOB. Next, gold nanorods for drift correction were immobilized on the surface via electrostatic interaction by incubating the gold nanorods for 2 min in FOB and afterward the sample was washed 3x with FOB. For DNA-PAINT measurements, surface passivation with ssDNA staples strands with 1 µM concentration in FOB was performed. The staples were incubated for 10 min and the sample was washed with FOB. Last, the buffer was exchanged for the experiment specific imaging buffer. The chamber was then sealed.

### pMINFLUX setup

The pMINFLUX setup is described in the original pMINFLUX publication^19^. For detailed information see the supporting information.

## Supplementary information

Supplementary Material GET-pMINFLUX

Data Video LPAINT
