# Supplementary material for: Combining pMINFLUX, graphene energy transfer and DNA-PAINT for nanometer precise 3D super-resolution microscopy
Source: Light Sci Appl. 2023 Mar 10;12:70. doi: 10.1038/s41377-023-01111-8 (PMC10006205; doi:10.1038/s41377-023-01111-8)
Supplement: Supplementary file 1 — Supplementary Material GET-pMINFLUX [file 41377_2023_1111_MOESM1_ESM.docx]

**Supporting information**

Combining pMINFLUX, Graphene Energy Transfer and DNA-PAINT for Nanometer Precise 3D Super-Resolution Microscopy

Jonas Zähringer^1^, Fiona Cole^1^, Johann Bohlen^1^, Florian Steiner^1,3^, Izabela Kamińska^1,2^, Philip Tinnefeld^1^

^1^ Department of Chemistry and Center for NanoScience, Ludwig-Maximilians-Universität München, Butenandtstr. 5-13 Haus E, 81377 München, Germany

^2^ Institute of Physical Chemistry Polish Academy of Sciences, Kasprzaka 44/52, 01-224 Warsaw, Poland

^3^ current address: Department of Physics, Ludwig-Maximilians-Universität München, Schellingstraße 4, 80799 München, Germany

Corresponding Author: [philip.tinnefeld@cup.uni-muenchen.de](mailto:philip.tinnefeld@cup.uni-muenchen.de)

# **Supplementary Section 1: Materials**

**1.1 Gold Nanorods**

Gold nanorods were used as fiducial markers for drift correction. The 900 nm long gold nanorods were fabricated following the protocol for long GNRs.^1^ After purification, transmission electron microscopy images were taken for characterization (Figure S1).


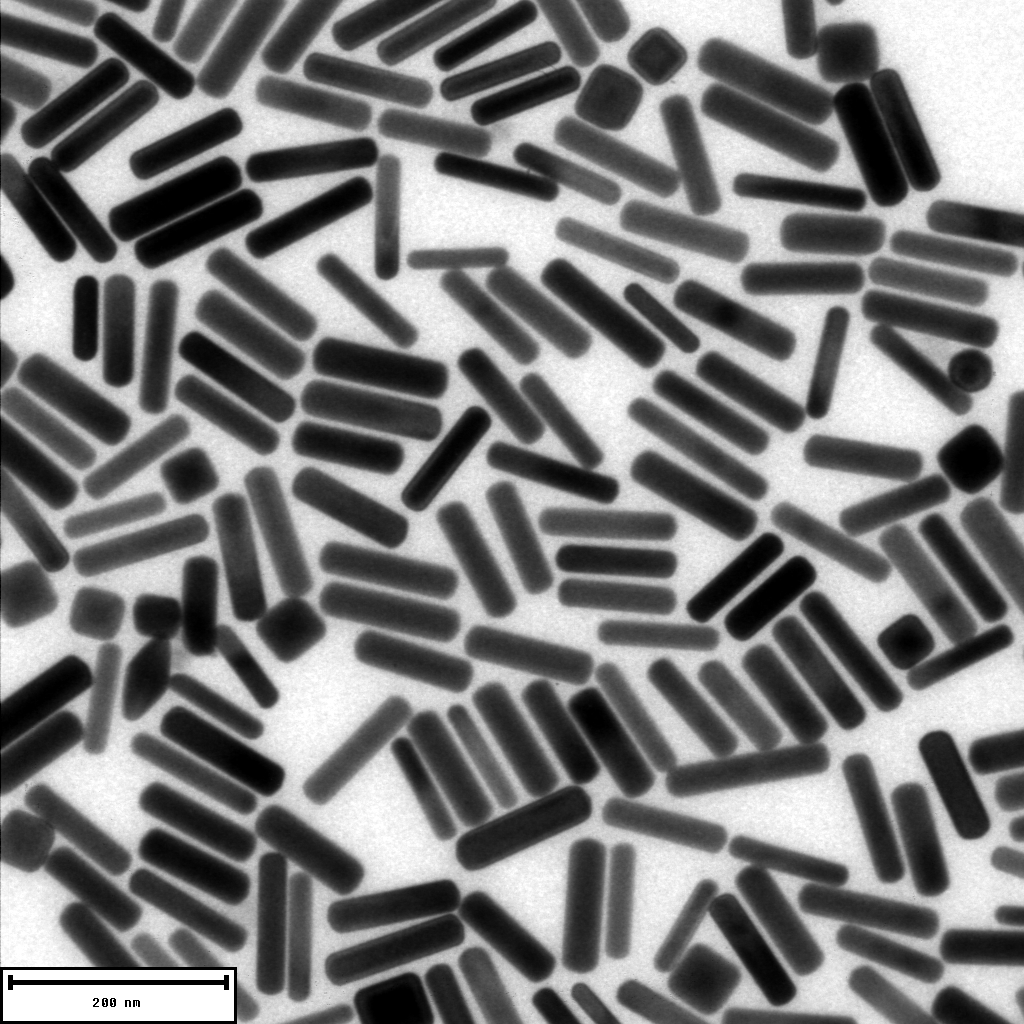


**Figure S1. TEM image of 900 nm long gold nanorods**

**1.2 pMINFLUX Setup**

The pMINFLUX setup is described in the original pMINFLUX publication.^2^ Depending on the excitation color, different optical elements such as filters, the vortex phase plate or polarization optics are used, however the beam path remains unchanged (Figure S2).


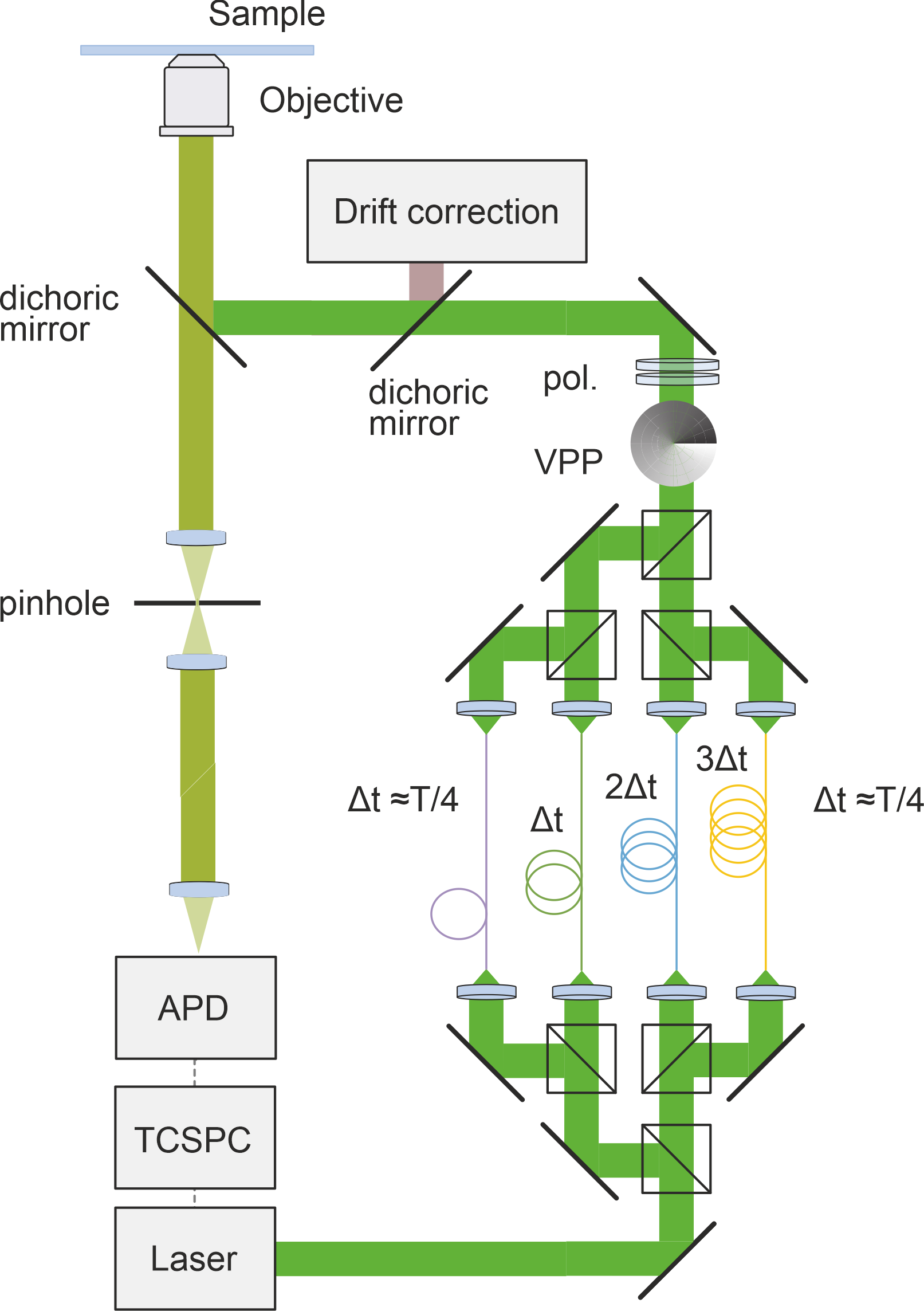


**Figure S2. pMINFLUX setup**. The pulsed laser is split into four beams and coupled to optical fibers, delaying the laserpulses as a function of the length of the fiber. The beams are recombined and doughnut shaped beams are created with a vortex phase plate and polarization optics. The beams are focused on the sample arranged in a triangular pattern with the fourth beam placed at the center of the triangle. For detection an APD is used together with a TCSPC unit.

**Excitation**. A supercontinuum laser (SuperK Fianium FIU-15, NKT Photonics GmbH, Germany) is used at 19.5 MHz repetition rate (*T*) as light source in combination with a tunable bandpass filter (SuperK VARIA, NKT Photonics GmbH, Germany) to select the desired wavelength range in the visible light spectrum. An additional clean-up filter (green: FLH532-10, Thorlabs GmbH, Germany, red: ZET 635/10, Chroma, USA) is used to further spectrally clean the excitation beam. Using a polarizing beam splitter cube (PBS251, Thorlabs GmbH, Germany) the light is split into two beams of orthogonal polarizations.

Each of the beams is further split by a non-polarizing 50:50 beam splitter cube (BS013, Thorlabs GmbH, Germany). This beam splitting system generates two pairs of beams with each pair sharing the orthogonal linear polarization. The resulting four laser beams are coupled into polarization maintaining single-mode fibers (PM-S405-XP, Thorlabs GmbH, Germany) of lengths 2.0 m, 4.6 m, 7.1 m and 9.7 m such that the time delay between the beams after the fiber is ~12.5 ns (= *T*/4). The four beams are collimated after the fibers with an achromatic lens (AC254-035-A, Thorlabs GmbH, Germany) and recombined by using three 50:50 beam splitter cubes (BS013, Thorlabs GmbH, Germany). The overlay of the beams can be adjusted to obtain the required arrangement of laser foci in the object plane. The axes of linear polarization are matched by turning the fiber out-couplers (Thorlabs GmbH, Germany). Subsequently, the linearly polarized laser beams pass a combination of a quarter- and a half-wave plate (WPQ05M-532 and WPH532M532, Thorlabs GmbH, Germany; for red: additional linear polarisator: LPVISC100-MP2, Thorlabs GmbH, Germany, RAC 5.2.10, B. Halle, Germany, WPQ05M-633, Thorlabs GmbH, Germany) to make them circularly polarized. A vortex phase plate (green: VPP, V-532-20-1, Vortex Photonics, Germany; red: VPP, V-633-20-1, Vortex Photonics, Germany) is then used to introduce the phase modulation necessary to generate the donut-shaped foci. The beams are guided into the back entrance of the microscope body (IX83, Olympus Deutschland GmbH, Germany), reflected on a dichroic mirror (ZT532/640rpc flat– STED, Chroma Technology Corp., USA) and focused with an objective (UPLSAPO100XO/1.4, Olympus Deutschland GmbH, Germany) onto the sample plane.

**Detection**. The fluorescence light is collected with the same objective and transmitted through the dichroic mirror, focused via an Olympus tube lens onto a pinhole (120 µm, Owis, Germany), collimated with an achromatic lens (AC254-150-A, Thorlabs GmbH, Germany). The beam is focused with a second achromatic lens (AC127-025-A, Thorlabs GmbH, Germany) to the chip of an avalanche photodiode (SPCM-AQRH-16-TR, Excelitas Technologies GmbH & Co. KG, Germany) after filtering the remaining scattered light from the laser with suitable interference optical filters (785 SP EdgeBasic, Semrock Inc., USA, green: 582/75 Brightline HC, Semrock Inc. USA, red: 700/75 ET Bandpass, Chroma, USA). The digital signal from the APD is sent to a TCSPC unit (HydraHarp 400, PicoQuant GmbH, Germany).

**Drift correction**. To measure and correct for sample drift during the measurement, the IR output of the variable bandpass filter is used. A beam of wavelength between 850 and 900 nm is selected with optical filters (875/50 bandpass, Edmund Optics GmbH), coupled into a single-mode fiber (780HP, Thorlabs GmbH, Germany), outcoupled and collimated. This beam is then split with a 50:50 beam splitter cube (BS014, Thorlabs GmbH, Germany) and combined again after inserting a lens system (ACN254-040-B, AC254-150-B, Thorlabs GmbH, Germany) into one of the two paths that focuses the beam to the back focal plane of the objective (dotted line) to create a widefield illumination at the sample plane. This beam is used for xy drift correction where the position of fiducial markers is localized during the measurement. The collimated IR beam is focused onto the sample plane at an oblique angle to achieve a z position-dependent spot at the detector and use this for z drift correction. Both IR beams are coupled to the main beam path via a dichroic mirror (ZT 785 SPXXR, Chroma Technology Corp., USA) and fed into the microscope to illuminate a region close, but not overlapping with the field of view used for MINFLUX. The reflected and backscattered light is split with an additional 50:50 beam splitter cube (BS014, Thorlabs GmbH, Germany) from the excitation IR beam and detected on a single CMOS camera (Zelux, Thorlabs GmbH, Germany) at different positions of the chip.

**Setup control**. The piezo stage (P733.3CD, Physik Instrumente (PI) GmbH &Co. KG, Germany) translates the sample in all three dimensions with a resolution of 0.3 nm when running in closed loop mode. All components of the setup including the piezo stage are controlled digitally and integrated via a custom version of the PyFLUX project. Further details and source-code of this control software version are available at <https://github.com/zaehringer-Jonas/pyflux>

**Alignment**. For MINFLUX measurements, the 4 vortex beams were aligned in a fixed triangular excitation beam pattern (EBP), with a *L* ≈ 100 nm.

# **Supplementary Section 2: Experiments**

**2.1 Reconvoluted Fluorescence Lifetime Fit**

For the fluorescence lifetime fit, the microtime histogram (also known as TCSPC histogram) was extracted for each localization so each time bin of the fluorescence trace (Figure S3 a). The microtime histogram was then rebinned such that the four decays overlay (Figure S3 b and c). The resulting single decay was then fitted with an IRF reconvoluted exponential fit using a least square minimization. To include background contributions, the fit has an additional background component. The background was determined after bleaching (Figure 1, 2 and 4) or for DNA-PAINT as no binding event was detected.


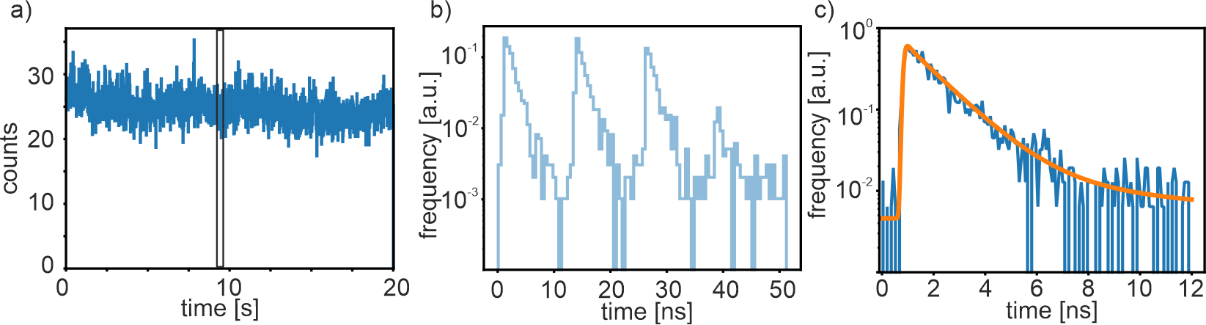


**Figure S3. Fitting of the fluorescence lifetime**. a) In the fluorescence intensity trace a time bin is selected. b) The microtime histogram of this time bin shows four peaks, corresponding to the four vortex beam excitations. c) The microtime histogram is rebinned such that it results in single decay. This is fitted using a IRF reconvoluted exponential fit (yellow line).

**2.2 Graphene MINFLUX Theory:**

The energy transfer efficiency from a dye molecule to graphene can be calculated from the equation:

$$\eta=1- \frac{\tau}{\tau_{0}}$$

where $\tau$ and $\tau_{0}$ stand for the fluorescence lifetime of a dye molecule immobilized on graphene or glass, respectively. At the same time, the energy transfer efficiency from an emitter to graphene scales with *d*^-4^, where d is the distance between the dye and graphene, and d_0_ is the distance of 50% energy transfer efficiency to graphene:

$$\eta=\frac{1}{1+\left( \frac{d}{d_{0}} \right)^{4}}$$

Based on both equations for the energy transfer efficiency, the distance *d* between the dye molecule and graphene is calculated: ^3^

$$d= d_{0}* \sqrt[4]{\frac{1}{\frac{\tau_{0}}{\tau}-1}}$$

with parameters for ATTO 647N: unquenched lifetime $\tau_{0}=4.2 ns$, and the 50% quenching height $d_{0}=18.5 nm$ and for ATTO 542 $\tau_{0}=3.4 ns$, and the 50% quenching height $d_{0}=17.7 nm$ and for Cy3b $\tau_{0}=2.6 ns$, and the 50% quenching height $d_{0}=17.7 nm$.^3^

In this article, instead of using *d* for the calculated distance values, we define the distance as *z*, to stay consistent with the three-axis *x, y, z* in a Cartesian coordinate system.

**2.3 Axial Precision**

For the theoretical errors of the *z* precision, first the error of fluorescence lifetime at a certain number of photons was calculated for a mono-exponential decay with constant background.^4^ Here, parameters were estimated according to the experiments with *N* ranging from 100 to 2000 photons, reconvoluted lifetimes $\tau$ from 1.0 to 3.8 ns, time window from 5 to 12 ns, 100 bins and SBRs from 2 – 16. The resulting variance of the fluorescence lifetime was then used to estimate the variance according to Gaussian error propagation:

$\sigma_{z}^{2}= \frac{1}{4} d_{0} {(\frac{\tau}{\tau_{0}- \tau})}^{-\frac{3}{4}} {(\frac{\tau_{0}}{\tau_{0}- \tau})}^{2}* \sigma_{\tau}^{2}$

The precision of *z* in dependence of the absolute value of *z* using quenched lifetimes and quenched SBR is plotted in Figure S4 with a fixed number of 1000 photons.


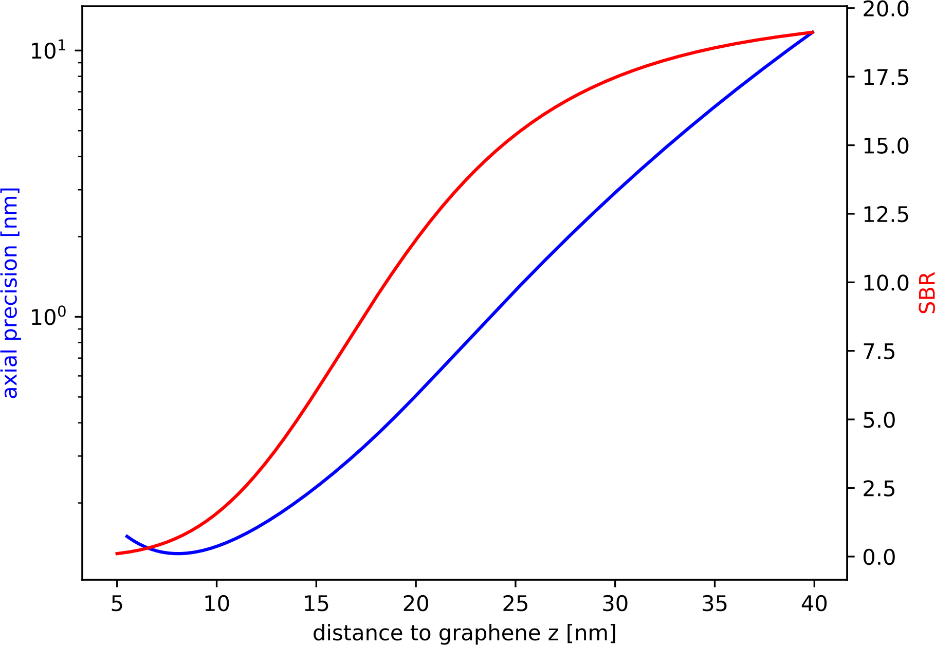


**Figure S4. Dynamic range of GET-pMINFLUX**. The theoretical value of SBR in red and the theoretical axial precision blue is plotted against the graphene distance.

On the one hand, with the condition of a SBR > 1, the lower end of GET-pMINFLUX will be limited to *z* > 8 nm. On the other hand, for isotropic precision, the axial precision should be similar to that of pMINFLUX. With 1000 photons this is reached at around 30-35 nm graphene distances. Hence for isotropic nanometer precise precision of GET-pMINFLUX, the graphene distance range is between 8 – 35 nm.

**2.4 DNA-PAINT measurement**

For DNA-PAINT measurements, a DNA origami structure (Figure S5 a) with in total 8 binding sites on its three faces were incorporated. The faces are labeled in Figure S4 b and their respective heights can be found in Table S2. On face A, 2 binding sites are spaced roughly 3 nm horizontally, on another face 2 binding sites are spaced 6 nm horizontally and on the third face there are 4 binding sites with 3 nm distance (Figure S5 b). Additionally, an internal dye (ATTO542) was incorporated to check correct orientation on graphene and graphene quality as well as to facilitate correct centering in the excitation beam pattern. For the DNA-PAINT measurements the sequence of T(TCC)_9_T was used as docking site sequence to increase the binding kinetics.^5^ The DNA-PAINT imager strand has the following sequence: AGGAGGA-ATTO542.


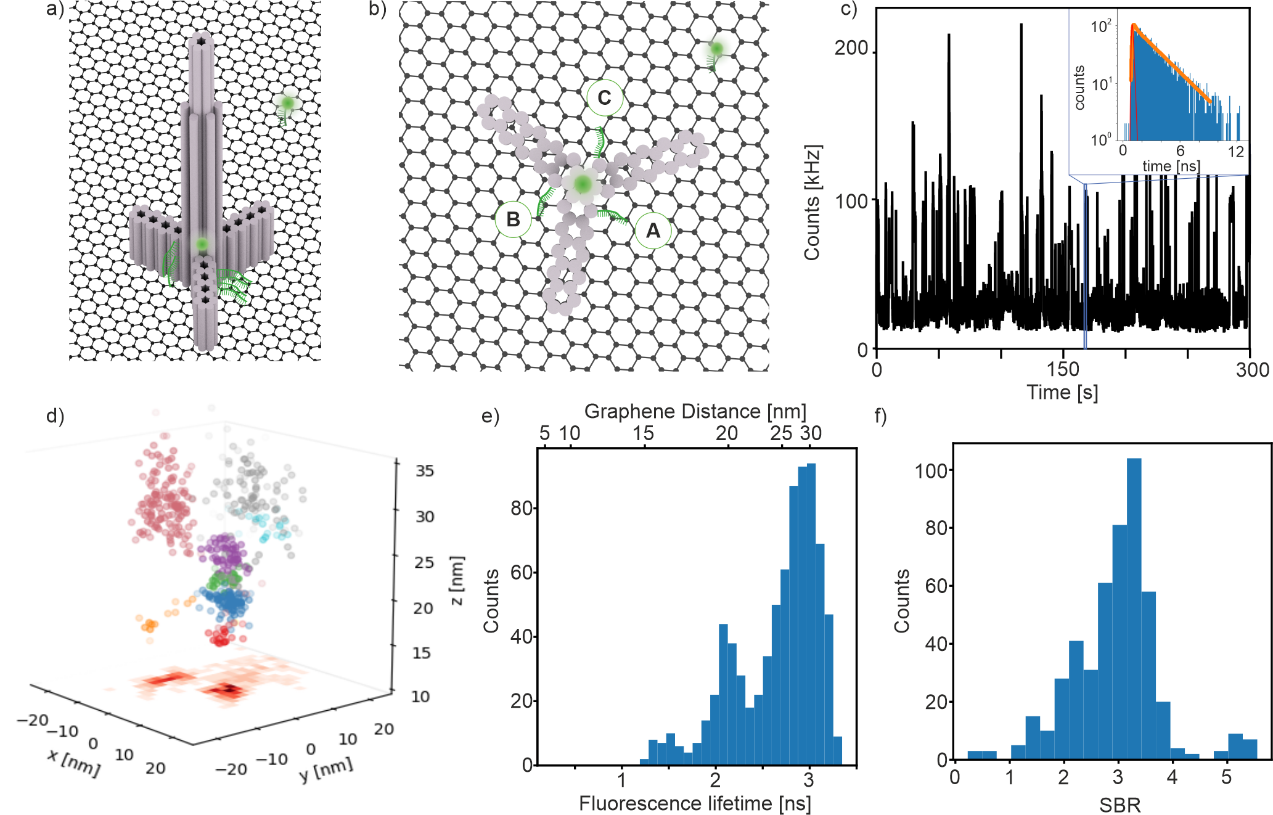


**Figure S*5*. GET pMINFLUX with DNA-PAINT**. a) Sketch of the DNA origami structure with indicated positions of permanent dye and docking strands. b) Top view with label for each face. Face A has 4 binding sites, face B and C have 2 binding sites each. The heights are indicated in Table S2. c) Trace of the fluorescence intensity showing binding events of the DNA-PAINT measurement. The inset shows the fitted fluorescence decay of such a segment together with the IRF. d) 3D localization plot of the trace. e) Histogram of the fluorescence lifetimes and the corresponding graphene distances. f) Histogram of the SBRs.

In the 300 s measurements (see intensity trace in Figure S5c), events were found using a fluorescence intensity threshold of 230 photons per 10 ms and a minimal event duration of 30 ms. To exclude double binding events, a maximum threshold of 1500 photons/10 ms was set. The extracted events were then binned into segments of 3500 photons. On the one hand, the segments were analyzed using a maximum likelihood estimator for xy localization. On the other hand, the fluorescence lifetime was extracted using a reconvoluted fit. The histogram of the fluorescence lifetimes is shown in Figure S5e, displaying several distinct peaks.

All events in the EBP are shown in figure S5d. Here, the two faces in front can be easily identified as face A and B and have xy distance of around 11 nm. Face A with its four docking sites is highlighted in Figure 3c by extracting all data points at the four binding sites in a diameter of 11 nm (radius = 5.5 nm). As the binding sites are different in their graphene distance also the quenching and the SBR is different. The histogram of the SBR (Figure S5f) shows distinct peaks in analogy to the histogram of fluorescence lifetimes.

**Table S2.** Designed Distances oft the DNA-PAINT docking sites to Graphene

| Face | Designed graphene distance of the docking site [nm] |
| --- | --- |
| A | 16.7  20  23  27.7 |
| B | 17.7  24.7 |
| C | 19  22.3 |

**2.5 Experimental DNA-PAINT precision**

As the DNA-PAINT binding sites are repetitive, rather long and furthermore are pointing parallel to the graphene surface. Assuming rapid molecular dynamics of the docking and imager strand within the integration time for one localization, the movement of the long binding sites average out for the axial orientation, but it has a potentially decreasing effect on the precision for xy localizations. Especially, as the docking strand has several binding positions, the localizations might be slightly broadened.

To estimate the effect of the length of the sequence the freely jointed chain model was used. The most extreme case of imager attaching at the different ends of the docking strand corresponds to a length of 21 nt ssDNA. With a Kuhn length of a chain segment of 1.5 nm for ssDNA^6^ results in an average end to end distance of the binding sites furthest apart of

$$\sqrt{\left\langle\left. R^{2} \right\rangle\right.}= \sqrt{\frac{21}{2}}*1.5 nm=4.9 nm$$

With the expectation of equal accessibility of all 8 binding sites, the resulting standard deviation is $\sigma_{x}=1.7 nm$.

In a DNA-PAINT experiment, the binding strands are protruding from the origami on the side. Due to steric hindrance of the tripod base of the DNA-origami the DNA-PAINT strand can only access about 120°. This is modeled with a simplified density function on 120° of a circle$: f\left( \alpha\right)=\frac{\cos\left( \alpha\right)}{\sqrt{3}}$ for $\alpha\in[-\frac{\pi}{3},\frac{\pi}{3} ]$ and $x=\sigma_{x} cos(\alpha)$ we get:

$$<\sigma_{x}> = \int_{-\frac{\pi}{3}}^{\frac{\pi}{3}} x f\left( \alpha\right)d\alpha= \int_{-\frac{\pi}{3}}^{\frac{\pi}{3}} \frac{\sigma_{x}*cos \left( \alpha\right)^{2}}{\sqrt{3}}d\alpha=\sigma_{x} (\frac{1}{4}+\frac{\pi}{3*\sqrt{3}})= 1.4 nm$$

The experimental precision is a convolution of the distribution of the spot and the MINFLUX precision. In the case of a MINFLUX precision of 1.5 nm an experimental precision of 2.1 nm would follow. This rough estimation agrees with the experimental DNA-PAINT MINFLUX data and indicates that the xy-precision of the pMINFLUX method itself is rather slightly better than the reported values.

# **3. DNA origami sequences**

**Table S3.** Core staples from the 5’ to the 3’ end for the pillar-shaped DNA origami structure.

| Staple ID | Sequence (5’ to 3’) |
| --- | --- |
| P1 | GAGAAGGCATCTGCAATGGGATAGGTCAAAAC |
| P2 | AACCGTGTCATTGCAACGGTAATATATTTTAAATGAAAGGGT |
| P3 | ATCGGTCAGATGATATTCACAAACCAAAAGA |
| P4 | GCTGGCATAGCCACATTATTC |
| P5 | CTGTATGGGATTACCGTTAGTATCA |
| P6 | CCATAATGCCAGGCTATCAAGGCCGGAGACATCTA |
| P7 | CTCATCGGGATTGAGTGAGCGAGTAACAACCCGTC |
| P8 | TAGCCAGCTTTCATCCAAAAATAAACGT |
| P9 | TAGCCTCAGAGCATACCCTGT |
| P10 | AATACCCCAACATTCATCAAAAATAATTCGCGTCT |
| P11 | GGCTAAAACTTCAGAAAAGTTTTGCGGGAGATAGAACC |
| P12 | CCCGGTTGATAAAGCATGTCAATC |
| P13 | ATCGATGCTGAGAGTCTACAAGGAGAGGGAACGCCAAAAGGA |
| P14 | GACAATTACGCAGAGGCATTTTCGAG |
| P15 | TAAGTTGGCATGATTAAAGAA |
| P16 | CCAATGTTTAAGTACGGTGTCCAAC |
| P17 | CGGAATAGAAAGGAATGCCTTGCTAAACAACTTTCAAC |
| P18 | GAGTTAAAAGGGTAATTGAGCGCTAATATCAGAGGAACTGAACACC |
| P19 | TTTAGCGATACCAACGCGTTA |
| P20 | TTTTTGCGGATGCTCCTAAAATGTTTAGATGAATTTTGCAAAAGAAGTT |
| P21 | AATAAAACGAACTATGACCCCACCAAGC |
| P22 | AATATCGTTAAGAGAGCAAAGCGGATTGTGAAAAATCAGGTCTTT |
| P23 | ATTACGAGATAAATGCCAGCTTTGAGGGGACGACGACAG |
| P24 | ACAACGCCTGTAGCATTTACCGTATAGGAAG |
| P25 | TTACCATTAGCAAGGCCTTGAATTAGAGCCAGCCCGACTTGAGC |
| P26 | CAGCAGCGCCGCTTGTTTATCAGCTTCACGAAAAA |
| P27 | CTTACGGAACAGTCAGGACGTTGGGAAGAAA |
| P28 | AGCTCTTACCGAAGCCCAATA |
| P29 | TATTACGAATAATAAACAAATCAGATATGCGT |
| P30 | CACGGCAACAATCCTGATATACTT |
| P31 | CATCGAGATAACGTCAAACATAAAAGAGCAAAAGAATT |
| P32 | CAAGCCCAATAGGAACCACCCTCACCCGGAA |
| P33 | CATTTCGCAAATGTCATCTGCGAACGAGAGATTCACAATGCC |
| P34 | GGCGCAGACGGTCAATCATCGAGACCTGCTCCATGTGGT |
| P35 | CAAACGGAATAGGAAACCGAGGAATAAGAAATTACAAG |
| P36 | ACCAACAAACCAAAATTAACAATTTCATTTGAATTACCGAGG |
| P37 | CATTTGAGATAACCCACGAAACAATG |
| P38 | AGGACAGATGAACGGTGTAACATAAGGGAACCGAAGAAT |
| P39 | TGGCTTTTTACCGTAGAATGGAAAGCG |
| P40 | GTTAAAGGAAAGACAGCATCTGCCTATTTAAGAGGCAGGAGGTTTA |
| P41 | AGTAGGTATATGCGTTATACA |
| P42 | CGAACACCAAATAAAATAGCAGCCAAGTTTGCCTTTAGCGTCAGA |
| P43 | GCGAAACAAAGTGTAAAACACATGGCCTCGATTGAACCA |
| P44 | AAGAAAGCTTGATACCGCCACGCATACAGACCAGGCGCTGAC |
| P45 | CTGAATATAGAACCAAATTATTTGCACGTAAAACAACGT |
| P46 | AGACAGCAGAAACGAAAGAGGAAATAAATCGAGGTGACAGTTAAAT |
| P47 | CGAGGGTACTTTTTCATGAACGGGGTCATAATGCCGAGCCACCACC |
| P48 | TAAAGCCTCCAGTACCTCATAGTTAGCG |
| P49 | AATATGCAACTACCATCATAGACCGGAACCGC |
| P50 | AGAAATCGTTAGACTACCTTTTTAAGGCGTTCTGACCTTTTTGCA |
| P51 | CTAAATCGGTCAGAATTAGCAAAATTAAGCAATAAAATAATA |
| P52 | AAATCAGCTCATTTTTTAACCATTTTGTTAAAATTCGCATTA |
| P53 | ATAGCGAGAGGCTATCATAACCAAATCCCAAAGAAAATTTCATCCTCAT |
| P54 | GAACTGGCTCATTACAACTTTAATCATTCTTGAGATTACTTA |
| P55 | ACGCGAGAGAAGGCCATGTAATTTAGGCCAGGCTTAATTGAGAATCGC |
| P56 | TAATATCAAAGGCACCGCTTCTGGCACT |
| P57 | TTTCCATGGCACCAACCTACGTCATACA |
| P58 | AAGACAAATCAGCTGCTCATTCAGTCTGACCA |
| P59 | CCGTAATCAGTAGCGACAGAATCTAATTATTCATTAAAAAGG |
| P60 | CTGGCATTAGGAGAATAAAATGAAGAAACGATTTTTTGAGTA |
| P61 | CGCGCCGCCACCAGAACAGAGCCATAAAGGTGGAA |
| P62 | TAGCCCGGAATAGGTGTAAGGATAAGTGCCGTCGA |
| P63 | AAGGCTCCAAAAGGAGCCTTTATATTTTTTCACGTGCTACAGTCACCCT |
| P64 | CAAAATCACCGGAACCAGAGCCAGATTTTGTCACAATCACAC |
| P65 | AATTGTGTCGAAATCCGCGGCACACAACGGAGATTTGTATCA |
| P66 | CCTCGTCTTTCCACCACCGGAACCGCCTCCCTCA |
| P67 | CCGTGTGATAAATAACCTCCGGCTGATG |
| P68 | CCCAGCTACAATGACAGCATTTGAGGCAAGTTGAGAAATGAA |
| P69 | TATTTAAATTGCAGGAAGATTG |
| P70 | AAGGGATATTCATTACCGTAATCTATAGGCT |
| P71 | ACCAGACCGGATTAATTCGAGC |
| P72 | AAGGCCTGTTTAGTATCATGTTAGCTACCTC |
| P73 | AGCAACAAAGTCAGAAATAATATCCAATAATCGGCTCAGGGA |
| P74 | TGAGTAAAGGATAAGTTTAGCTATATCATAGACCATTAGATA |
| P75 | GAGTCTGGATTTGTTATAATTACTACATACACCAC |
| P76 | TTCGGTCCCATCGCATAGTTGCGCCGACATGCTTTCGAGGTG |
| P77 | CGTGTCAAATCACCATCTAGGTAATAGATTT |
| P78 | GGAACCATACAGGCAAGGCAAATCAAAAAGACGTAGTAGCAT |
| P79 | ATTTGGAAGTTTCATGCCTCAACATGTTTTA |
| P80 | AATTTCTTAAACCCGCTTAATTGTATCGTTGCGGGCGATATA |
| P81 | GAGCATTTATCCTGAATCAAACGTGACTCCT |
| P82 | TTATAAGGGTATGGAATAATTCATCAATATA |
| P83 | TAACGACATTTTTACCAGCGCCAAAGAAAGTTACCAGAACCCAAA |
| P84 | AAAGATTACAGAACGGGAGAAGGAAACGTCACCAATGAAACCA |
| P85 | GCTGTAGTTAGAGCTTAATTG |
| P86 | AGTTTCCAACATTATTACATTATAC |
| P87 | GGGATATTGACGTAGCAATAGCTAAGATAGC |
| P88 | AACAAGAGCCTAATGCAGAACGCGC |
| P89 | AGTTTATTGTCCATATAACAGTTGATTC |
| P90 | TATTGAAAGGAATTGAGGTAG |
| P91 | AATAGAAAAAAATAAACGTCTGAGAGGAATATAAGAGCAACACTATGAT |
| P92 | TCGTGCCGGAGTCAATAGTGAATTTGCAGAT |
| P93 | TTAGTTTGAGTGCCCGAGAAATAAAGAAATTGCGTAGAGATA |
| P94 | TTGGTAGAACATTTAATTAAGCAAC |
| P95 | TAACATCCAATAAATGCAAAGGTGGCATCAACATTATGAAAG |
| P96 | TAAGTTTACACTGAGTTTCGT |
| P97 | AGAACTTAGCCTAATTATCCCAAGCCCCCTTATTAGCGTTTGCCA |
| P98 | ACCGCCACCCTCAGAACCCGTACTCTAGGGA |
| P99 | TTAGCCCTGACGAGAAACACCAGAAATTGGGGTGAATTATTTTAA |
| P100 | ATAAAGTCTTTCCTTATCACT |
| P101 | ATTTCCTGATTATCAGATGATGGCTTTAAAAAGACGCTAAAA |
| P102 | ACATAAGTAGAAAAATCAAGAAGCAAAAGAAGATGTCAT |
| P103 | TTCATCGGCATTTTCGGTCATATCAAAA |
| P104 | GAACCGCCACCCTCCATATCATACC |
| P105 | ACTAATGCCACTACGAATAAA |
| P106 | CAAGCCGCCCAATAGCAAGTAAACAGCCATATTATTTTGCCATAAC |
| P107 | TGAAAATCCGGTCAATAACCTAAATTTTAGCCTTT |
| P108 | CCTCGTTTACCAGAAACCAAA |
| P109 | CAAATTATTCATTTCAATTACCTGAGTA |
| P110 | ATTTCAACCAAAAATTCTACTAATAGTTAGTTTCATTTGGGGCGCGAGC |
| P111 | AGGCTTGCGAGACTCCTCAAGAGAAAAGTATTCGGAAC |
| P112 | AATATTCATTGAATCCATGCTGGATAGCGTCCAAT |
| P113 | CTAGTCAGTTGGCAAATCAACAGTCTTTAGGTAGATAACAAA |
| P114 | TATGACTTTATACATTTTTTTTTAATGGAAACAGTACACCGT |
| P115 | ACTAAAGAGCAACGTGAAAATCTCCACCCACAACTAAAGGAA |
| P116 | TTGCGAATAATATTTACAGCGGAGTGAGGTAAAATTTTGAGG |
| P117 | CCGACTTGTTGCTAAAATTTATTTAGTTCGCGAGAGTCGTCTTTCCAGA |
| P118 | ATTGTTATCTGAGAAGAAACCAGGCAAAGCGCCATTCGTAGA |
| P119 | AGTACCGCATTCCACAACATGTTCAGCCTTAAGGTAAAGTAATTC |
| P120 | AAACTCACAGGAACGGTACGCCAGTAAAGGGGGTGAGGAACC |
| P121 | CGCTTTCCAGTTAGCTGTTTAAAGAACGT |
| P122 | GGCGAAGCACCGTAATAACGCCAGGGTTTTCCCAGTCATGGG |
| P123 | TTTACCAGTCCCGGCCTGCAGCCCACTACGGGCGCACCAGCT |
| P124 | GGCAACACCAGGGTCTAATGAGTGAGCTCACAACAATAGGGT |
| P125 | GAAGGAGCGGAATTATCATCATATATCATTTACATAGCACAA |
| P126 | CGCGCTACAGAGTAATAAAAGGGACATTCTGATAGAACTTAG |
| P127 | GTAATTAATTTAGAATCTGGGAAGGGCGATCGGTGCGGCAAA |
| P128 | GGATGTGGTTTGCCCCAGCAG |
| P129 | GCCAGCAGTTGGGCGCAAATCAGGTTTCTTGCCCTGCGTGGT |
| P130 | TATCAGCAACCGCAAGAATGCCAATGAGCCTGAGGATCTATC |
| P131 | GAGAACAATATACAAAATCGCGCAGAGGCGATTCGACAAATCCTTTAAC |
| P132 | GTAAAACGACGGCCCATCACCCAAATCAGCGC |
| P133 | ACGGGCCGATAATCCTGAGAAGTGTTTTTATGGAGCTAACCG |
| P134 | TGCTAAATCGGGGAGCCCCCGATTTAGAGCTAGCAGAACATT |
| P135 | ACGCGGTCCGTTTTTGGGTAAGTGA |
| P136 | GCGTCCACTATTCCTGTGTGAAATGCTCACTGCC |
| P137 | CGTACTATGGTAACCACTAGTCTTTAATGCGCGAACTGAATC |
| P138 | AGAATTTTAGAGGAAAACAATATTACCGCCAGCTGCTCATTT |
| P139 | TTGGGCGGCTGATTTCGGCAAAATCCCT |
| P140 | TGGTGGTTGTTCCAGTTTGGAACA |
| P141 | AGTCGCCTGATACTTGCATAACAGAATACGTGGCACAGCTGA |
| P142 | TGCTGATTGCCGTTGTCATAAACATCGGGCGG |
| P143 | TGAGTGTTCCGAAAGCCCTTCACCGCCTAGGCGGTATTA |
| P144 | TGAGCAAATTTATACAGGAATAACATCACTTGCCTGAGTCTT |
| P145 | CCTGCGCTGGGTGGCGAGAAAGGAAGGGAAGGAGCGGGGCCG |
| P146 | CGTACAGGCCCCCTAACCGTCCCCGGGTACCGAGCGTTC |
| P147 | TTTAGATTCACCAGTCACACGACCGGCGCGTGCTTTCCCAGA |
| P148 | CCCCGCTAGGGCAACAGCTGGCGAAAGGGGGATGTGCTTATT |
| P149 | TCACAGCGTACTCCGTGGTGAAGGGATAGCTAAGAGACGAGG |
| P150 | TGCGTGTTCAGGTTGTGTACATCG |
| P151 | AGGGAGCCGCCACGGGAACGGATAGGCGAAAGCATCAGCACTCTG |
| P152 | AAGAAAGCGCTGAACCTCAAATATTCTAAAGGAAAGCGTTCA |
| P153 | AGCGCAGCTCCAACCGTAATCATGGTCACGGGAAACCT |
| P154 | CCTCATCACCCCAGCAGGCCTCTTCGCTATTACGCCAGTGCC |
| P155 | GTCGCGTGCCTTCGAATTGTCAAAG |
| P156 | TTCGGGGTTTCTGCCAGGCCTGTGACGATCC |
| P157 | AGAGAAAATCCAGAGAGTTGCAGCAAATC |
| P158 | TGCCATCCCACGCAGGCAGTTCCTCATTGCCGTTTTAAACGA |
| P159 | GCCCGAGTACGAGCCGGAAGC |
| P160 | GAGGCCAAGCTTTGAATACCAAGTACGGATTACCTTTTCAAA |
| P161 | ACGTAAGAATTCGTTCTTAGAAGAACTCAAACTATCGGATAA |
| P162 | TAAAACCGTTAAAGAGTCTGTCCATCCAGAAACCACACAATC |
| P163 | ACGAGCGGCGCGGTCAGGCAAGGCGATTAAGTTGGGTAAAAC |
| P164 | TTTTCCAGCATCAGCGGGGCTAAAGAACCTCGTAGCACGCCA |
| P165 | CAAAGCACTAGATAGCTCCATTCAGGCTGCGCAACTGTCTTG |
| P166 | ATTGCGTTGCTGTTATCCGCTCACAATTCCAAACTCACTTGCGTA |
| P167 | GAGAGATAGACTTTACGGCATCAGA |
| P168 | TGACCGCGCCTTAATTTACAATATTTTTGAATGGCTATCACA |
| P169 | CCTAATTTAACAAACCCTCAATCAATATCTGATTCGCTAATC |
| P170 | TTAACTCGGAATTAGAGTAAATCAATATATGTGAGTGATTCT |
| P171 | ATGAAGGGTAAAGTTCACGGTGCGGCCATGCCGGTCGCCATG |
| P172 | ACATAAAGCCCTTACACTGGTCGGGTTAAATTTGT |
| P173 | AAATGCGGAAACATCGGTTTTCAGGTTTAACGTCAGATTAAC |
| P174 | GTCGCAGAAAAACTTAAATTTGCC |
| P175 | GAATTCGTCTCGTCGCTGGGTCTGCAATCCATTGCAACACGG |
| P176 | GCGAAAATCCCGTAAAAAAAGCCGTGGTGCTCATACCGGCGTCCG |
| P177 | CTTGTAGAACGTCAGCGGCTGATTGCAGAGTTTTTCGACGTT |
| P178 | TCATACATTTAATACCGATAGCCCTAAAACATCGAACGTAAC |
| P179 | TACGGCTGGAGGTGCGCACTCGTCACTGTTTGCTCCCGGCAA |
| P180 | AAATGACGCTAAATGGATTATTTACATTGGCGAATACCTGGA |
| P181 | AACAACAGGAAGCACGTCCTTGCTGGTAATATCCAGAAACGC |
| P182 | TGCATTAATGAGCGGTCCACGCTCACTGCGCCACGTGCCAGC |
| P183 | ACCTGACGGGGAAAGCCGGCGAACCAAGTGTCTGCGCGTTGC |
| P184 | CCAGCCTCCGATCCTCATGCCGGA |
| P185 | GCTGGTCTGGTCAGGAGCCGGAATCCGCCGTGAACAGTGCCA |
| P186 | GCGAATCAGTGAGGCCACCGAGTAGTAGCAACTGAGAGTTGA |
| P187 | GGCCAACGCGCGGGGAGGGCCCTGTGTTTGA |
| P188 | AGCTTTCAGAGGTGGCGATGGCCAGCGGGAAT |
| P189 | ATTAGCGGGGTTTTGCTCAGTACCAGGCTGACAACAAGCTG |
| P190 | TGCCCGTATAAACAGTGTGCCTTCTGGTAA |
| P191 | AGAAAACGAGAATGACCATAAATCTACGCCCCTCAAATGCTTTA |
| P192 | ATAACTATATGTAAATGCTTAGGATATAAT |
| P193 | AGGAATCATTACCGCGTTTTTATAAGTACC |
| P194 | GATTAGAGAGTACCTTAACTCCAACAGG |
| P195 | CCTTAAATCAAGATTAGCGGGAGGCTCAAC |
| P196 | GCATGTAGAAACCAATCCATCCTAGTCCT |

**Table S4.** Staples from the 5’ to the 3’ end for the pillar-shaped DNA origami structure with extensions for pyrene-modified staples binding.

| Sequence (5’ to 3’) | Function | Replace |
| --- | --- | --- |
| ATATTTCCTCTACCACCTACATCACTAATTAGCGGGGT TTTGCTCAGTACCAGGCTGACAACAAGCTG | External labeling with pyrene | P189 |
| ATATTTCCTCTACCACCTACATCACTAAGAAAACGAG AATGACCATAAATCTACGCCCCTCAAATGCTTTA | External labeling with pyrene | P190 |
| ATATTTCCTCTACCACCTACATCACTAATAACTATATG TAAATGCTTAGGATATAAT | External labeling with pyrene | P191 |
| ATATTTCCTCTACCACCTACATCACTAGCATGTAGAAA CCAATCCATCCTAGTCCTG | External labeling with pyrene | P192 |
| ATATTTCCTCTACCACCTACATCACTATGCCCGTATAA ACAGTGTGCCTTCTGGTAA | External labeling with pyrene | P193 |
| ATATTTCCTCTACCACCTACATCACTAAGGAATCATTA CCGCGTTTTTATAAGTACC | External labeling with pyrene | P194 |
| ATATTTCCTCTACCACCTACATCACTAGATTAGAGAGT ACCTTAACTCCAACAGG | External labeling with pyrene | P195 |
| ATATTTCCTCTACCACCTACATCACTACCTTAAATCAA GATTAGCGGGAGGCTCAAC | External labeling with pyrene | P196 |
| GTGATGTAGGTGGTAGAGGAAATAT-**pyrene** | Pyrene at 3’ | - |

**Table S5.** Staples from the 5’ to the 3’ end for the pillar-shaped DNA origami structure for Distance determination from fluorescence lifetimes.

| Sequence (5’ to 3’) | Function | Replace |
| --- | --- | --- |
| AGACAGCAGAAACGAAAGAGGAAATAAATCGAGGTG ACAGTTAAAT- **ATTO647N** | Dye ATTO647N at 3’ (11.6 nm) | P46 |
| CATTTGAGATAACCCACGAAACAATG-**ATTO647N** | Dye ATTO647N at 3’ (15.9 nm) | P37 |
| AAGGGATATTCATTACCGTAATCTATAGGCT-**ATTO647N** | Dye ATTO647N at 3’ (23.4 nm) | P70 |
| ATTGTTATCTGAGAAGAAACCAGGCAAAGCGCCATTCGTAGA-**ATTO647N** | Dye ATTO647N at 3’ (30 nm) | P118 |

For the Experiment the staples of pillar-shaped DNA origami structure: 3, 4, 6, 13, 15, 23, 30, 43, 45, 49, 50, 61, 64, 66, 68, 75, 79, 82, 90, 91, 92, 102, 105, 157, 157, 167 were replaced with the following:

**Table S6.** Staples from the 5’ to the 3’ end for the pillar-shaped DNA origami structure for DNA-PAINT experiments

| CCAGAACAGAGCCATAAAGGTGGAATAAGTTGGCATGATTAAAGAAAATAGAAA T TCCTCCTCCTCCTCCTCCTCCTCCTCCT | DNA-PAINT binding site |
| --- | --- |
| AACTACCATCATAGACCGGAATCTGGATTTGTTATAA T TCCTCCTCCTCCTCCTCCTCCTCCTCCT | DNA-PAINT binding site |
| TTATAAGGGTATGGAATAATTCATCAATATAATCCT T TCCTCCTCCTCCTCCTCCTCCTCCTCCT | DNA-PAINT binding site |
| CGCCACATAAGTAGAAAAATCAAGAAGCAAAAGAAGATGATGGC T TCCTCCTCCTCCTCCTCCTCCTCCTCCT | DNA-PAINT binding site |
| ACAATGACAGCATTTGAGGCAGGTCAGATGATATTC T TCCTCCTCCTCCTCCTCCTCCTCCTCCT | DNA-PAINT binding site |
| AGAAATCGTTAGGAATATAAGAGCA T TCCTCCTCCTCCTCCTCCTCCTCCTCCT | DNA-PAINT binding site |
| CCATAATGCCAGGCTATCAAGGCCGGAGACATCTAGCTGG T TCCTCCTCCTCCTCCTCCTCCTCCTCCTCAT | DNA-PAINT binding site |
| TCGTGCCGGAGTCAATAGTGAATTTGCAGATAAAAGGAAT T TCCTCCTCCTCCTCCTCCTCCTCCTCCT | DNA-PAINT binding site |
| TATTGAAAGGAATTGAGGAA T TCCTCCTCCTCCTCCTCCTCCTCCTCCT | DNA-PAINT binding site |
| AAAATAAACGTCTGAGAGACTACCTTTTTAAGGC | Exchange staple |
| TTACTACATACACCACCCTCGTCTTTCCACCACCGGAACCGCCTCCCTCA | Exchange staple |
| CGCAAAGACACCACGGCAACA | Exchange staple |
| CAAAATCACCGGAACCAGAGCCAGATTTTGTCA | Exchange staple |
| GATATACTTCTGAATATAGAACCAAATTAT | Exchange staple |
| ATAGCGAGAGGCTATCATAACCAAATCCCAAAGAAAATTTCATCCTCAT | Exchange staple |
| CTCGATTGAACCAGAGCCGCCGCGCCGCCA | Exchange staple |
| GCGAAACAAAGTGTAAAACACTCAT | Exchange staple |
| AGTTTCCAACATTATTACATT | Exchange staple |
| TTGCACGTAAAACAACGT | Exchange staple |
| ACAAACCAAAAGAATACACTAATGCCACTACGAATAAA | Exchange staple |
| GTTCTGACCTTTTTGCACCCAGCT | Exchange staple |
| AGCCACATTATTCATCAGTTGAGAAATGAA | Exchange staple |
| TTCATGCCTCAACATGTTTTAAATATGC | Exchange staple |
| TACGAGATAAATGCCAGCTTTGAGGGGACGACGACAG | Exchange staple |
| CAAGAGAATCGATGCTGAGAGTCTACAAGGAGAGG | Exchange staple |
| ACACTATGATATTTGGAAGT | Exchange staple |
| ACCGGAATCTGGATTTGTTATAA | Exchange staple |
| TTCATGCCTCAACATGTTTTAAATATGCAACTACCATCATAGT - ATTO542 | Internal Dye |

|  |
| --- |
|  |

Table S7. Core staples from the 5’ to the 3’ end for the L-shaped DNA origami structure for LPAINT L197-L252 were left out in the experiments with the L-shaped DNA origami structure labeled with 42 pyrene molecules.

| staple ID | Sequence (5’ to 3’) |
| --- | --- |
| L1 | ATCCAGAACAATATTAGTCCATCAGGAACGGT |
| L2 | CGTGCCTGTTCTTCGCATCCAGCGCCGGGTTA |
| L3 | ATAATCAGAAAAGCCCAACATCCACTGTAATA |
| L4 | CATAGGTCTGAGAGACAAATCGTCGAATTACC |
| L5 | ATTGCCCTTCACCGCCCCAGCTGCTTGCGTTG |
| L6 | TTCGTAATCATGGTCATCCATCAGTTATAAGT |
| L7 | CCCGCCGCGCTTAATGAAAGCCGGCGAACGTG |
| L8 | AGGCGAAAATCCTGTTGTCTATCACCCCCGAT |
| L9 | GCTGCGCAACTGTTGGCAGACCTATTAGAAGG |
| L10 | CTGCAACAGTGCCACGTATCTGGTAGATTAGA |
| L11 | AACAGAGGTGAGGCGGCAGACAATTAAAAGGG |
| L12 | AAATCCCGTAAAAAAACGTTTTTTGGACTTGT |
| L13 | GGCTTAGGTTGGGTTAAGCTAATGATTTTCGA |
| L14 | TATTTTGTTAAAATTCGGGTATATATCAAAAC |
| L15 | GTATAAGCAAATATTTTAGATAAGTAACAACG |
| L16 | CCAGCCAGCTTTCCGGGTAATGGGGTAACAAC |
| L17 | GGGGTCATTGCAGGCGGGAATTGACTAAAATA |
| L18 | TGTTGCCCTGCGGCTGATCAGATGCAGTGTCA |
| L19 | GGAAACCAGGCAAAGCGTACATAAGTGAGTGA |
| L20 | CTCTCACGGAAAAAGAACGGATAAAAACGACG |
| L21 | ATCGGCAAAATCCCTTACGTGGACTCCAACGT |
| L22 | TCAAATCACCATCAATACGCAAGG |
| L23 | GCAGTTGGGCGGTTGTCCAGTTATGGAAGGAG |
| L24 | CTTCTGACCTAAATTTGCAGAGGCCAGAACGCAATTTACG |
| L25 | ATCAAACTTAAATTTCTGGAAGGGCCATATCA |
| L26 | TATCATTTTGCGGAACATCCTGATATAAAGAA |
| L27 | GACCGTGTGATAAATACAAATTCT |
| L28 | TGATTGCTTTGAATACAAACAGAATGTTTGGA |
| L29 | GCCGGGCGCGGTTGCGCCGCTGACCCCTTGTG |
| L30 | GTACTATGGTTGCTTTTTTAGACACGCAAATT |
| L31 | GGGCCTCTTCGCTATTACGTTGTACCTCACCG |
| L32 | GCAGCAAGCGGTCCACAAGTGTTTTGAGGCCA |
| L33 | AACGTTATTAATTTTACAACTAATCAGTTGGC |
| L34 | GAAATTGTTATCCGCTCACATTAAATTAATGA |
| L35 | CCAGCTTACGGCTGGAAACGTGCCCGTCTCGT |
| L36 | GCAGAGGCGAATTATTTTTCATTTGCTATTAA |
| L37 | CATTGCCTGAGAGTCTTTATGACCATAAATCATTTCATTT |
| L38 | CTAGCTGATAAATTAACAGTAGGG |
| L39 | AAATCAGCTCATTTTTGTGAGCGAATAGGTCA |
| L40 | TATTTTTGAGAGATCTGCCATATTTCCTCTACTCAATTGA |
| L41 | CAGGAAAAACGCTCATACCAGTAAATTTTTGA |
| L42 | ACAGTTGAGGATCCCCAGATAGAACTGAAAGC |
| L44 | AGAAACAGCTTTAGAAGGAAGAAAAATCTACGATTTTAAGCATATAAC |
| L45 | GCACCCTCCGTCAGGTACGTTAGTAAATGAATAGTTAGCGTCAATCAT |
| L46 | AGTTGATTAGCTGAAAAGAGTACCTTTAATTGTTAATTCGGACCATAA |
| L47 | CTCAAATGTTCAGAAATGGAAGTTTCACGCGCATTACTTCAACTGGCT |
| L48 | TTTCATCGAATAATATCCAGCTACAATACTCCAGCAATTTCTTTACAG |
| L49 | TGCTCATTCTTATGCGTTAATAAAACGAACTATATTCATTGGCTTTTG |
| L50 | GGCACCAAAACCAAAAGTAAGAGCAACACTATAGCAACGTAAATCGCC |
| L51 | AAGGGAACCGGATATTCACTCATCTTTGACCCGTAATGCCATCGGAAC |
| L52 | ATATTCACCGCCAGCATTGACAGGCAAAATCA |
| L53 | CGGAATCTCAGGTCTGTTTTAAATATGCATGCGAACGAATCATTG |
| L54 | AAAGACAAATTAGCAAGTCACCAATGAAACCA |
| L55 | TCGATAGCAGCACCGTAAAATCACGTTTTGCT |
| L56 | TGAATTACCAGTGAATGGAATTACGAGGCATATAGCGAGAGAATCCCC |
| L57 | TAGTTGCCAGTTGCGGGAGGTTTTGAAGATCAATAA |
| L58 | GCCCCCTGGTGTATCACCGTACTC |
| L59 | AATAAGTTAGCAAAAACGCAATAATAACGAGAATTAAAAGCCCAA |
| L60 | CAAAAGAATAAAATACCCAGCGATTATACCAAGCGCGAA |
| L61 | TTTTCATCGGCATATTGACGGCACCACGG |
| L62 | GGGGCGCGCCCAATTCACTAAAGTACGGTGTCACGAGAATAGCTTCAA |
| L63 | CCGGCAAATCGGCGAAGTGGTGAAGGGATAG |
| L64 | ATCAAAAAGTCATAAAACGGAACAACATTATCAACTTTAGTAGAT |
| L65 | TTAGTTTGCCTGTTTAGGTCATTTTTGCGGATAGGAAGCCGACTATTA |
| L66 | GCGAGAAAAGGGATGACGAGCACGTATAACGTGCTTTTCACGCTGAAGAAAGC |
| L67 | CCCTGAACAAATAAGAAACGCGAGGCGTT |
| L68 | CTGAGGCCAACGGCTACAGAGGTTTCCATT |
| L69 | ACATTCTGAAGAGTCTCCGCCAGCAGCTCGAA |
| L70 | AAATCAACACGTGGCATCAGTATTCTCAATCC |
| L71 | TTATACTTAGCACTAAAAAGTTTGTGCCGCCA |
| L72 | CCAACATGACGCTCAATGCCGGAGGAAATACC |
| L73 | CCGGAACCGCAAGAAAGCAATAGCTATCTTACTCACAATCCGATTGAG |
| L74 | GTAAGAATAGTTGAAACTTTCGCAAACACCGC |
| L75 | GCCAGTGCGATTGACCCACCGCTTCTGGTGCC |
| L76 | AGGAAACCGAGGACGTAGAAAAAGTACCG |
| L77 | CTGCGCGGCTAACTCACAATTCCACACAACATACGAGTACCGGGGCTCTGTGGGTGTTCAG |
| L78 | AATTACATAGATTTTCAATAACGGATTCGCC |
| L79 | ATAACCTTATCAACAAAAATTGTATAACCTCC |
| L80 | CCAGAATGGAGCCGCCAATCAAGTTTGCC |
| L81 | TTTTTTAATGCACGTACAAGTTACCCATTCAG |
| L82 | CATTATACGGTTTACCCATAACCCTCGAAATACAATGTTTAAACAGGG |
| L83 | CTTTTGCGTTATTTCAATGATATTCAACCGTT |
| L84 | GACAGATGGACCTTCATCAAGAGCCCTGAC |
| L85 | ACAAGAAATAGGAATCCCAATAGCAAGCAAATATAGCAGCATCCTGAA |
| L86 | AAATTATTTGGAAACAGCCATTCGAAAATCGC |
| L87 | CACTCATGAAACCACCTTAAATCAAGATTGAGCGTCTTTTTGTTT |
| L88 | GCCTAATTATCATATGATAAGAGATTTAGTTAATTTCAT |
| L89 | GAGGGTAGTTGCAGGGTGCTAAACAACTTTCACGCCTGGAAAGAG |
| L90 | AGAGCCGCAAACAAATGAGACTCCTCAAGAGATTAGCGGGCAGTAGCA |
| L91 | ATTGCGTTTAACAACATTTCAATTACCTGAGCAAAAGGGAGAAACAGGTTTAAGATGATGG |
| L92 | CCACCCTCTGTTAGGAAGGATCGTCTTTCCAGCAGACGATTATCAGCT |
| L93 | GCCAGTACGTTATAAGGCGTTAAATAAGAATAAACACAAAT |
| L94 | CAATTCATATAGATAATAAATCCTTTGCCCG |
| L95 | GCCGTCACAATATAAAAGAAACCACCAGAAGGAGCGGACTCGTATTACATTTGTCAAATAT |
| L96 | TACCAGTAACGCTAACAGTTGCTATTTTGCACCCCATCCT |
| L97 | GTCGAAATCCGCGACCTGCTCCACCAACTTTTAGCATTC |
| L98 | GTCCACTAAACGCGCGGACGGGCAACAGCTG |
| L99 | AACCGTTTCACACGGGAAATACCTACATTTTGACGCTAAACTATCACTTCTTTAACAGGAG |
| L100 | CGCTGGCACCACGGGAGACGCAGAAACAGCGG |
| L101 | CAAATCGTCAGCGTGGTGCCATCCCACGCAA |
| L102 | GCCGATTAAGGAAGGGCGCGTAACCACCACA |
| L103 | TGTAGCTCAACATTTACCCTCGAAAGAC |
| L104 | GAGAAACATTTAATTTTACAGGTAGAAAG |
| L105 | TTGAGTAAGCCACCCTCAGAACCG |
| L106 | TTAGAGCTATCCTGAGGCTGGTTTCAGGGCGC |
| L107 | TTCACCAGGTAGCAATGGCCTTGCTGGTAAT |
| L108 | CGCTCACTATCAGACGGTCCGTGAGCCTCCTC |
| L109 | ATTCATATCAGTGATTTGGCATCAGGACGTTGTAACATAAACCAGACG |
| L110 | GGAGGGAAGAGCCAGCAATCAGTAGCGACAGACCAGAACCGCCTC |
| L111 | AACGTCAATAGACGGGGAATACCCAAAAGAACAAGACTCCGTTTTTAT |
| L112 | TGTACTGGTAATAAGTTCAGTGCC |
| L113 | TTCAAATTTTTAGAAAAAACAGGAGCAAACAAGAGAATCGATGAAGGGTGAGATATTTTA |
| L114 | TAATAAGAAGAGCCACCCTTATTAGCGTTTGCCATTCAACAATAGAAA |
| L115 | TTCTGAAACATGAAAGTGCCGGCCATTTG |
| L116 | CAAACCCTTTAGTCTTACCAGCAGAAGATAA |
| L117 | AAACGGGGTTTTGCTACATAACGCCAAAAAAGGCTTGTAATCTTG |
| L118 | TGGAGCCGGCCTCCGGGTACATCGACATAAAA |
| L119 | CCGAGTAAGCCAACAGGGGTACCGCATTGCAA |
| L120 | ACAAGAACCGAACTGATGTTACTTAGCCGGAAAAGACAGCACTACGAA |
| L121 | AGAACGTTAACGGCGTAATGGGTAAAGGTTTCTTTGCGTCGGTGGTGCTGGTCTTGCCGTT |
| L122 | GGAGCCTTCACCCTCAGAGCCACC |
| L123 | CCCCCTGCGCCCGCTTTAGCTGTTTCCTGTGT |
| L124 | TGCGGGATAGCAGCGACGAGGCGCAGAGAAACGGCCGCGGTAACGATC |
| L125 | TAATAGTATTCTCCGTGCATTAAATTTTTGTT |
| L126 | CACATCCTCAGCGGTGGTATGAGCCGGGTCAC |
| L127 | CACAGACATTTCAGGGATCTCCAAAAAAAAGGTTCTTAAAGCCGCTTT |
| L128 | CCATTACCAAGGGCGACATCTTTTCATAGGCAGAAAGAATAGGTTGAG |
| L129 | ATGAGTGACCTGTGCAGTTTCTGCCAGCACG |
| L130 | AAGCGCATAAATGAAACAGATATAGAAGGCTTAGCAAGCCTTATTACG |
| L131 | ATAAAAATATCGCGTTCTCCTTTTGATAAGAGCTATAT |
| L132 | ATCGGCCTTAAAGAATAAATCAAAAGAATAGCCCGAGACCAGTGAGGGAGAGGGGTGCCTA |
| L133 | CCTGCAGCCATAACGGGGTGTCCAGCATCAGC |
| L134 | ATGGCTACAATCAACTGAGAGCCAGCAGCAAATGAAAAACGAACCTAATGCGCTTGGCAGA |
| L135 | TACAGGCATTAAATTAACCAATAGGAACGCCATCAAAGTCAATCAGAATTAGCCTAAATCG |
| L136 | CCGTCGGAGTAGCATTCAAAAACAGGAAGATT |
| L137 | GTTTTCCCGTAGATGGCAGGAAGATCGCACT |
| L138 | GCCTGTTTGCTTCTGTTACCTTTTAACGTTAA |
| L139 | AAACGGCGCAAGCTTTGAAGGGCGATCGGTGC |
| L140 | TACCGATAGTTGCGCTTTTTCA |
| L141 | CAGTACCATTAGTACCCAGTGCCCGTATAAATTGATGAATTAAAG |
| L142 | CAACTAATGCAGACAGAGGGGCAATACTG |
| L143 | ACCCTCATGCCCTCATTTTCTGTATGGGATTTAGTTAAAGCAGCTTGA |
| L144 | ATAAACAATCCCTTAGTGAATTTATCAAAAT |
| L145 | CCTCAGAGCACAAGAAGAAAAGTAAGCAG |
| L146 | CAGTATGTTTATTTTGCGAAGCCCTTTTTAATTGAGTTCTGAACA |
| L147 | CGGGAAACGAAAAACCTGATGGTGGTTCCGAA |
| L148 | CTTAATTGAGACCGGAAACAGGTCAGGATTAGAGGTGGCA |
| L149 | TCATCAACAAGGCAAATATGTACCCCGGTTG |
| L150 | TGCTTTCGAGGTGAATCTCCAAAA |
| L151 | AGCATGTACGAGAACAATCCGGTATTCTAAGAACGATTTTCCAGA |
| L152 | TCTTACCATAAAGCCATAATTTAGAATGGTTTAGGGTAGC |
| L153 | CGTTGAAAATAGCAAGCCCAATA |
| L154 | GTTGTACCACCCTCATAAAGGCCGGAGACAG |
| L155 | GAAACAACGCGGTCGCCGCACAGGCGGCCTTTAGTGACTTTCTCCACGTACAGACGCCAGG |
| L156 | CAAAGGGCCTGTCGTGTGGCCCTGAGAGAGTT |
| L157 | TTAATTTCATGTTCTATAACTATATGTAAATGCTGATGTCAATAGAATCCTTGACAAAATT |
| L158 | AGCGAACCAGAAGCCTGGAGAATCACAAAGGCTATCAGGT |
| L159 | CGTTGGTAGTCACGACGCCAGCTGGCGAAAGGGGGATATCGGCCTGCGCATCGGCCAGCTT |
| L160 | GGAACCCAAAACTACAAACAGTTTCAGCG |
| L161 | AGGAGGTGGCGGATAAGTATTAAGAGGCTAAATCCTCTACAGGAG |
| L162 | GGAATTAGGTAAATTTTCGGTCATAGCCCCACCGGAACCACCACC |
| L163 | TCTTTAGGCTGAATAATGCTCATTAGTAACAT |
| L164 | TGCGAATAATAATCGACAATGTTCGGTCG |
| L165 | ACGCCAGATGACGGGGCGCCGCTAGCCCCAGC |
| L166 | TAAAGTTTAGAACCGCTAATTGTATCGCGGGGTTTAAGTTTGGCCTTG |
| L167 | ATTATAGCGTCGTAATAGTAAAATGTTTTTT |
| L168 | TTTTTTTTTTTTAAAACTAG |
| L169 | TTTTTGCCTGAGTAGAAGAA |
| L170 | TTTTGATTAAGACGCTGAGA |
| L171 | TTTTGGCGCATAGGCTGGCTAACGGTGTTAAATTGT |
| L172 | TTTGCGTATTGGGCGCTTTT |
| L173 | TAGTCAGAAGCAAAGCGGATTTT |
| L174 | TTTTCGCAAATGGTCAATAAACCATTAGATGC |
| L175 | TTTTTTGCATCAAAAGCCTGAGTAATTTT |
| L176 | TTTTCCATATTATTTATCCCAATCCAAAGTCAGAGA |
| L177 | GAAAGGAGCGGGCGCTAGGTTTT |
| L178 | ATATATATAAAGCGACGACATCGGCTGTCTTTCCTTATCATTTTT |
| L179 | TCAGCAGCAACCGCAATTTT |
| L180 | TTTTGTTTCGTCACCAGTACTGTACCGTAAT |
| L181 | TTTTCTTTACAAACAATTCG |
| L182 | TTTTACCGTTCCAGTAAGCGTCATACATGGCTTCAGTTAAT |
| L183 | TTTTGGAATTTGTGAGAGAT |
| L184 | AGAGCAAATCCTGTCCAGATACCGACAAAAGGTAATTTT |
| L185 | ATACGCAAAGAAAATTATTCATTAAAGGTGAATTTT |
| L186 | TTAATTAAACCATACATACATAAAGGTGGCAATTTT |
| L187 | CTGATAGCCCTAAAACTTTT |
| L188 | TTTTATTGGGCTTGAGATGGCCAGAACGATT |
| L189 | CAGATGAATATACAGTTTTT |
| L190 | TTTTCGGGCCGTTTTCACGG |
| L191 | CCGTGCATCTGCCAGTTTTT |
| L192 | TTTTGCTAATATCAGAGAGATAACCCCGCCACCGCG |
| L193 | ACAAAGTATGAGGAAGCTTTGAGGACTAAAGATTTT |
| L194 | TTTCGACTTGATCGAGAGGGTTGATATAAGTATTTT |
| L195 | TTTTCCCTCAGAGCCACCACCCTCAGAAAGCGCTTA |
| L196 | GAGCCGATATAACAACAACCATCGCCCTTTTTTT |

Table S8. Staples from the 5’ to the 3’ end for the L-shaped DNA origami structure with extensions for pyrene-modified staple binding.

| Sequence (5’ to 3’) | Function | Replace |
| --- | --- | --- |
| ATATTTCCTCTACCACCTACATCACTAATCCAGAACAA TATTAGTCCATCAGGAACGGT | External labeling with pyrene | L1 |
| ATATTTCCTCTACCACCTACATCACTACGTGCCTGTTC TTCGCATCCAGCGCCGGGTTA | External labeling with pyrene | L2 |
| ATATTTCCTCTACCACCTACATCACTAATAATCAGAAA AGCCCAACATCCACTGTAATA | External labeling with pyrene | L3 |
| ATATTTCCTCTACCACCTACATCACTACATAGGTCTGA GAGACAAATCGTCGAATTACC | External labeling with pyrene | L4 |
| ATATTTCCTCTACCACCTACATCACTAATTGCCCTTCA CCGCCCCAGCTGCTTGCGTTG | External labeling with pyrene | L5 |
| ATATTTCCTCTACCACCTACATCACTATTCGTAATCAT GGTCATCCATCAGTTATAAGT | External labeling with pyrene | L6 |
| ATATTTCCTCTACCACCTACATCACTACCCGCCGCGCT TAATGAAAGCCGGCGAACGTG | External labeling with pyrene | L7 |
| ATATTTCCTCTACCACCTACATCACTAAGGCGAAAATC CTGTTGTCTATCACCCCCGAT | External labeling with pyrene | L8 |
| ATATTTCCTCTACCACCTACATCACTAGCTGCGCAACT GTTGGCAGACCTATTAGAAGG | External labeling with pyrene | L9 |
| ATATTTCCTCTACCACCTACATCACTACTGCAACAGTG CCACGTATCTGGTAGATTAGA | External labeling with pyrene | L10 |
| ATATTTCCTCTACCACCTACATCACTAAACAGAGGTGA GGCGGCAGACAATTAAAAGGG | External labeling with pyrene | L11 |
| ATATTTCCTCTACCACCTACATCACTAAAATCCCGTAA AAAAACGTTTTTTGGACTTGT | External labeling with pyrene | L12 |
| ATATTTCCTCTACCACCTACATCACTAGGCTTAGGTTG GGTTAAGCTAATGATTTTCGA | External labeling with pyrene | L13 |
| ATATTTCCTCTACCACCTACATCACTATATTTTGTTAA AATTCGGGTATATATCAAAAC | External labeling with pyrene | L14 |
| ATATTTCCTCTACCACCTACATCACTAGTATAAGCAAA TATTTTAGATAAGTAACAACG | External labeling with pyrene | L15 |
| ATATTTCCTCTACCACCTACATCACTACCAGCCAGCTT TCCGGGTAATGGGGTAACAAC | External labeling with pyrene | L16 |
| ATATTTCCTCTACCACCTACATCACTAGGGGTCATTGC AGGCGGGAATTGACTAAAATA | External labeling with pyrene | L17 |
| ATATTTCCTCTACCACCTACATCACTATGTTGCCCTGC GGCTGATCAGATGCAGTGTCA | External labeling with pyrene | L18 |
| ATATTTCCTCTACCACCTACATCACTAGGAAACCAGGC AAAGCGTACATAAGTGAGTGA | External labeling with pyrene | L19 |
| ATATTTCCTCTACCACCTACATCACTACTCTCACGGAA AAAGAACGGATAAAAACGACG | External labeling with pyrene | L20 |
| ATATTTCCTCTACCACCTACATCACTAATCGGCAAAAT CCCTTACGTGGACTCCAACGT | External labeling with pyrene | L21 |
| ATATTTCCTCTACCACCTACATCACTATCAAATCACCA TCAATACGCAAGG | External labeling with pyrene | L22 |
| ATATTTCCTCTACCACCTACATCACTAGCAGTTGGGCG GTTGTCCAGTTATGGAAGGAG | External labeling with pyrene | L23 |
| ATATTTCCTCTACCACCTACATCACTACTTCTGACCTA AATTTGCAGAGGCCAGAACGCAATTTACG | External labeling with pyrene | L24 |
| ATATTTCCTCTACCACCTACATCACTAATCAAACTTAA ATTTCTGGAAGGGCCATATCA | External labeling with pyrene | L25 |
| ATATTTCCTCTACCACCTACATCACTATATCATTTTGC GGAACATCCTGATATAAAGAA | External labeling with pyrene | L26 |
| ATATTTCCTCTACCACCTACATCACTAGACCGTGTGAT AAATACAAATTCT | External labeling with pyrene | L27 |
| ATATTTCCTCTACCACCTACATCACTATGATTGCTTTG AATACAAACAGAATGTTTGGA | External labeling with pyrene | L28 |
| ATATTTCCTCTACCACCTACATCACTAGCCGGGCGCGG TTGCGCCGCTGACCCCTTGTG | External labeling with pyrene | L29 |
| ATATTTCCTCTACCACCTACATCACTAGTACTATGGTT GCTTTTTTAGACACGCAAATT | External labeling with pyrene | L30 |
| ATATTTCCTCTACCACCTACATCACTAGGGCCTCTTCG CTATTACGTTGTACCTCACCG | External labeling with pyrene | L31 |
| ATATTTCCTCTACCACCTACATCACTAGCAGCAAGCGG TCCACAAGTGTTTTGAGGCCA | External labeling with pyrene | L32 |
| ATATTTCCTCTACCACCTACATCACTAAACGTTATTAA TTTTACAACTAATCAGTTGGC | External labeling with pyrene | L33 |
| ATATTTCCTCTACCACCTACATCACTAGAAATTGTTAT CCGCTCACATTAAATTAATGA | External labeling with pyrene | L34 |
| ATATTTCCTCTACCACCTACATCACTACCAGCTTACGG CTGGAAACGTGCCCGTCTCGT | External labeling with pyrene | L35 |
| ATATTTCCTCTACCACCTACATCACTAGCAGAGGCGAA TTATTTTTCATTTGCTATTAA | External labeling with pyrene | L36 |
| ATATTTCCTCTACCACCTACATCACTACATTGCCTGAG AGTCTTTATGACCATAAATCATTTCATTT | External labeling with pyrene | L37 |
| ATATTTCCTCTACCACCTACATCACTACTAGCTGATAA ATTAACAGTAGGG | External labeling with pyrene | L38 |
| ATATTTCCTCTACCACCTACATCACTAAAATCAGCTCA TTTTTGTGAGCGAATAGGTCA | External labeling with pyrene | L39 |
| ATATTTCCTCTACCACCTACATCACTATATTTTTGAGA GATCTGCCATATTTCCTCTACTCAATTGA | External labeling with pyrene | L40 |
| ATATTTCCTCTACCACCTACATCACTACAGGAAAAACG CTCATACCAGTAAATTTTTGA | External labeling with pyrene | L41 |
| ATATTTCCTCTACCACCTACATCACTAACAGTTGAGGA TCCCCAGATAGAACTGAAAGC | External labeling with pyrene | L42 |
| GTGATGTAGGTGGTAGAGGAAATAT-pyrene | Pyrene at 3’ | - |

**Table S9.** Staples from the 5’ to the 3’ end for the L-shaped DNA origami structure for L-PAINT

| Sequence (5’ to 3’) | Function | Replace |
| --- | --- | --- |
| GGCACCAAAACCAAAAGTAAGAGCAACACTATAGCA ACGTAAATCGCCTTTTTTTTTCGGGCATTTA-**Cy3B** | Pointer-Cy3B at 3’ | L43 |
| AACGAATCATTGTGAATTACCTTTTTTAAATGCC | Lower binding site | L49 |
| GGCACCAAAACCAAAAGTAAGAGCAACACTATAGCA ACTTTTAAATGC | Middle binding site | L50 |
| AGCGTCAATCATAAGGGAACCGGTTTTAAATGCC | Upper binding site | L51 |
| GCACCCTCCGTCAGGTACGTTAGTAAATGAATAGTT | Exchange staple | L45 |
| TGCTCATTCAGTGAATGGAATTACGAGGCATATAGCG AGAGAATCCCC | Exchange staple | L49 |
| ATATTCACTCATCTTTGACCCGTAATGCCATCGGAAC | Exchange staple | L51 |
| ATATTCACCGCCAGCATCGATAGCAGCACCGTAAAAT CACGTTTTGCT | Exchange staple | L52 |
| CGGAATCTCAGGTCTGTTTTAAATATGCATGCG | Exchange staple | L53 |
| GTAAATCGCCAAAGACAAATTA | Exchange staple | L54 |
| GCAAGTCACCAATGAAACCATTGACAGGCAAAATCA | Exchange staple | L55 |
| ATGCGTTAATAAAACGAACTATATTCATTGGCTTTTG | Exchange staple | L56 |

REFERENCES

1. Wang S, Xi W, Cai F, Zhao X, Xu Z, Qian J*, et al.* Three-photon luminescence of gold nanorods and its applications for high contrast tissue and deep in vivo brain imaging. *Theranostics* 2015, **5**(3)**:** 251-266.

2. Masullo LA, Steiner F, Zahringer J, Lopez LF, Bohlen J, Richter L*, et al.* Pulsed Interleaved MINFLUX. *Nano Lett* 2021, **21**(1)**:** 840-846.

3. Kaminska I, Bohlen J, Rocchetti S, Selbach F, Acuna GP, Tinnefeld P. Distance Dependence of Single-Molecule Energy Transfer to Graphene Measured with DNA Origami Nanopositioners. *Nano Letters*; 2019. pp. 4257-4262.

4. Thiele JC, Nevskyi O, Helmerich DA, Sauer M, Enderlein J. Advanced Data Analysis for Fluorescence-Lifetime Single-Molecule Localization Microscopy. *Front Bioinform* 2021, **1:** 740281.

5. Strauss S, Jungmann R. Up to 100-fold speed-up and multiplexing in optimized DNA-PAINT. *Nat Methods* 2020, **17**(8)**:** 789-791.

6. Nickels PC, Wunsch B, Holzmeister P, Bae W, Kneer LM, Grohmann D*, et al.* Molecular force spectroscopy with a DNA origami-based nanoscopic force clamp. *Science* 2016, **354**(6310)**:** 305-307.
